# Say My Name: Understanding the Power of Names, Correct Pronunciation, and Personal Narratives

**DOI:** 10.15766/mep_2374-8265.11284

**Published:** 2022-11-29

**Authors:** Salma Dali, Anaid Atasuntseva, Megha Shankar, Eve Ayeroff, Malorie Holmes, Christina Johnson, Abdullah Sulieman Terkawi, Beth Beadle, Joon Chang, Kathleen Boyd, Tamara Dunn

**Affiliations:** 1 Fellow, Department of Pediatrics, Division of Pediatric Hospital Medicine, University of California, San Francisco; 2 Clinical Instructor, Department of Psychiatry and Behavioral Sciences, Division of Child and Adolescent Psychiatry, Stanford University; 3 Assistant Professor of Medicine, Department of Medicine, Division of General Internal Medicine, University of California, San Diego; 4 Adjunct Instructor, Department of Pediatrics, Division of Neonatology, University of California, San Francisco; 5 General Nephrologist, Hypertension and Kidney Consultants of Georgia; 6 Maternal Fetal Medicine Fellow, Department of OB/GYN, Stanford University; 7 Clinical Assistant Professor, Department of Anesthesiology, Perioperative, and Pain Medicine, Stanford University; 8 Professor, Department of Radiation Oncology, Stanford University; 9 Clinical Assistant Professor, Department of Medicine, Division of Pulmonary, Allergy, and Critical Care, Stanford University; 10 Clinical Assistant Professor, Department of Pediatrics, Stanford University; 11 Clinical Assistant Professor, Department of Medicine, Division of Hematology, Stanford University

**Keywords:** Names, Pronunciation, Microaggression, Case-Based Learning, Anti-racism, Diversity, Equity, Inclusion

## Abstract

**Introduction:**

Names are a reflection of identity and often have personal meaning. The chronic mispronunciation of names can undermine one's identity and be experienced as a microaggression. This workshop aims to provide historical context for names as well as resources for correct name pronunciation.

**Methods:**

We developed a 60-minute interactive virtual workshop with didactics, small-group sharing of personal experiences, and case discussions. We used an anonymous postworkshop survey to evaluate workshop effectiveness.

**Results:**

We presented the workshop at one local academic conference and two local educational conferences to learners of all levels from medical students to faculty. We collected postworkshop survey results from 78 participants of diverse racial and ethnic backgrounds. Participants reported learning historical context, ways to ask about correct name pronunciation, correcting name mispronunciation, documenting pronunciation, and sources for applications to practice. The main barriers to implementing workshop lessons included personal and structural factors.

**Discussion:**

This workshop effectively fills an educational gap by addressing the importance of correct name pronunciation in order to provide a more inclusive environment for clinicians and patients alike.

## Educational Objectives

By the end of this workshop, participants will be able to:

1. Examine the importance of name pronunciation in identity affirmation.
2. Illustrate the historical instances of racism that contribute to name mispronunciation.
3. Employ tools to engage in productive conversation around name pronunciation.
4. Apply name affirmation tools to clinical setting, medical education, and workplace.

## Introduction

At their foundation, names are a reflection of identity. Names often have meaning to an individual and are frequently associated with a racial, ethnic, cultural, or religious group. The mispronunciation of names can undermine one's identity, similar to misgendering. Chronic mispronunciation can lead to feeling marginalized, not accepted or included, and undervalued and can be experienced as a microaggression.^1,2^

The links between names and racial discrimination are deeply rooted in history. Enslaved people were given European-sounding slave names to replace their African ones, and the last name imposed was oftentimes the name of the slave owner. After emancipation, many formerly enslaved people changed their names as a way of reclaiming their identities.^3,4^

The education world has had the led the way in focusing on name pronunciation as an equity and inclusion issue. The classroom is a place where children have formative experiences in relation to their names, and the way their teachers and peers react to their names can set the stage for their academic careers. Students of color have recounted instances in which teachers renamed students with more traditionally American-sounding names or were unable to pronounce names and thus approximated them to names of objects. These experiences were identified as racial microaggressions. The cumulative effect from experiencing repeated microaggressions can lead to a shift in self-perception and cause one to feel inferior, also known as internalized racial microaggressions.^2^

Similar experiences have been highlighted in business and health care. Names can influence a person's school performance, job opportunities, and even arrest record, among other outcomes. Evidence shows that that Western-sounding or Whitened names on resumes have higher job callback rates when compared to racial/ethnic minority sounding names.^5,6^

The profound impact of correct or incorrect name pronunciation points to a necessity for more education in this area, particularly for training on how to correctly pronounce names. While there is currently one publication in *MedEdPORTAL* describing a workshop that teaches medical students how to ask patients about identity, intersectionality, and resilience,^7^ there are no *MedEdPORTAL* publications that introduce the importance of and an approach to properly stating someone's name in various academic settings—clinical, education, or workplace.

## Methods

### Facilitators

A diverse group of clinical scholars including medical residents, fellows, and faculty supervisors of different specialties developed, implemented, and evaluated this workshop. Initially, eight presenters who were part of the Leadership Equity and Advancing Diversity program at Stanford facilitated the workshop. The number of facilitators was adapted depending on audience size and facilitator availability. Facilitators self-assigned roles ahead of time, including presenting workshop segments, sharing personal experience, leading small groups, and facilitating large-group discussion. All facilitators were familiar with the entire workshop; therefore, each facilitator was able to lead different segments of the workshop if others were unavailable. Facilitators had to be comfortable leading small-group discussions with learners of different levels and backgrounds, although no specific prior knowledge or experience was required to facilitate the workshop.

### Audience

The target audience for the workshop was broad and included learners of all levels, such as medical students, medical residents, physician assistants, nurse practitioners, clinical faculty, researchers, staff, and community members. While the workshop was presented in settings that mostly captured medical audiences, it was also applicable to learners in nonmedical fields, including teachers, business professionals, and lawyers.

### Workshop

We developed the workshop following Kern's six-step model for curriculum development.^8^ For steps 1 and 2, we conducted a general needs assessment by doing a literature review focusing on name mispronunciation and identity. For step 3, we developed goals and objectives for the session based on the literature review and suggestions from the workshop authors. For step 4, we chose interactive educational strategies, including a PowerPoint presentation, polls, videos, case discussion, and large-group debrief. For step 5, we presented the workshop virtually at various educational conferences targeted towards learners. For step 6, we created a postworkshop evaluation based on the workshop learning objectives and content.

Using feedback from the postworkshop evaluation, we modified the workshop between iterations. We initially developed and presented it as a 75-minute workshop and adapted it to 60 minutes to meet audience needs. Based on participant feedback, we kept videos and allowed time for discussion in all iterations, as these were valued components of the workshop. We also asked facilitators to provide examples in the small-group discussions if participants did not engage. This allowed the space to remain safe given that sensitive topics might be raised and some participants might not feel comfortable sharing. We randomly divided participants into virtual small groups with six to 10 participants per group. Ideally, there were one to two facilitators per small group, although, due to the number of participants, some small groups did not have any facilitators.

Applying the Miller framework, learners in this workshop progressed through levels 1–3 of the pyramid of assessment.^9^ At the first level, they directly observed examples of name pronunciation and correcting mispronunciation. At the second level, they demonstrated learning in small groups by sharing examples of correct pronunciation of their own names. At the third level, they engaged in case presentations to directly apply their knowledge.

We started the workshop by introducing facilitators and workshop objectives. At the beginning of the workshop, facilitators shared their personal experience with name pronunciation in small groups to foster vulnerability. Then, we asked participants to share their personal experiences as well. Facilitators led a didactic portion, including historical context on name pronunciation, literature on effects of name mispronunciation, and tools for name pronunciation and correcting mispronunciation. The didactics were interspersed with a poll asking participants whether they had mispronounced someone's name and/or had their name mispronounced. We also included topical videos on name pronunciation to break up the didactic portion and engage the audience. We then placed participants back into their same small groups to discuss two case scenarios with corresponding questions. We preassigned the two case scenarios to small groups to allow both cases to be discussed. Each group had the option to discuss both cases depending on time. We concluded the workshop with large-group debrief of cases and a workshop evaluation.

### Timeline and Materials

#### Preworkshop

• Preparation: Facilitators reviewed the PowerPoint slides and facilitator guide. Either case 1 or case 2 was assigned to each small-group facilitator for breakout room 2.
• Materials: PowerPoint slides (Appendix A), facilitator guide (Appendix B).

#### Workshop

• Materials: PowerPoint slides (Appendix A), facilitator guide (Appendix B), participant handout (Appendix C).
• Outline: 75-minute version.
  ○ Introduction and objectives: 5 minutes.
  ○ Breakout room 1—personal experience: 10 minutes.
  ○ Didactics: 35 minutes total.
    ▪ Background: 3 minutes.
    ▪ Poll: 1 minute.
    ▪ Historical context: 3 minutes.
    ▪ Video 1: 3 minutes.
    ▪ Names and racism: 4 minutes.
    ▪ Video 2: 1 minute.
    ▪ Tools for pronunciation: 15 minutes.
    ▪ Application Zoom exercise: 1 minute.
    ▪ Application to health care: 4 minutes.
  ○ Breakout room 2—case discussions: 15 minutes.
  ○ Large-group debrief and evaluations: 10 minutes.
• Outline: 60-minute version.
  ○ Introduction and objectives: 5 minutes.
  ○ Breakout room 1—personal experience: 10 minutes.
  ○ Didactics: 30 minutes total.
    ▪ Background: 3 minutes.
    ▪ Poll: 1 minute.
    ▪ Historical context: 3 minutes.
    ▪ Video 1: 3 minutes.
    ▪ Names and racism: 4 minutes.
    ▪ Video 2: 1 minute.
    ▪ Tools for pronunciation: 10 minutes.
    ▪ Application Zoom exercise: 1 minute.
    ▪ Application to health care: 4 minutes.
  ○ Breakout room 2—case discussions: 10 minutes.
  ○ Large-group debrief and evaluations: 15 minutes.

### Evaluation and Analysis

As the workshop concluded, participants were asked to complete the anonymous postworkshop evaluation (Appendix D) online; a link to the survey was posted in the virtual workshop chat, and a QR code was also provided. The evaluation used a combination of items rated on a 5-point Likert scale (1 = *strongly disagree,* 5 = *strongly agree*) and open-ended questions to evaluate the effectiveness of the workshop.

We used descriptive statistics to analyze demographic data and summarized participant open-ended responses.

### Institutional Review Board

This study was submitted for review by the Stanford University Institutional Review Board and was determined not to meet the definition of human subject research (Protocol Number: 61348, approval date: May 12, 2021).

## Results

We presented this workshop at one local academic conference (Annual Stanford Medicine Diversity & Inclusion Forum, May 2021) and two local educational conferences (Stanford Department of Neurology All-Staff Meeting, November 2021; Stanford Pediatrics Noon Educational Conference, November 2021). Seventy-eight people completed the postworkshop evaluation out of 147 total participants.

Participants comprised 3% medical students, 9% residents, 4% fellows, 9% clinician researchers, 20% clinician faculty, 6% business, 11% education, and 38% other roles, including staff, administration, undergraduate or graduate students, chiropractors, and counselors. Participants also had diverse racial and ethnic backgrounds; of those who answered, 1% identified as American Indian or Alaskan Native; 23% as Asian; 7% as Black or African American; 5% as Hispanic, LatinX, or of Spanish origin; 57% as White or Caucasian; and 7% as other, including two or more ethnic groups, American, Filipino, Iranian, and White immigrant (Table 1).

**Table 1.**
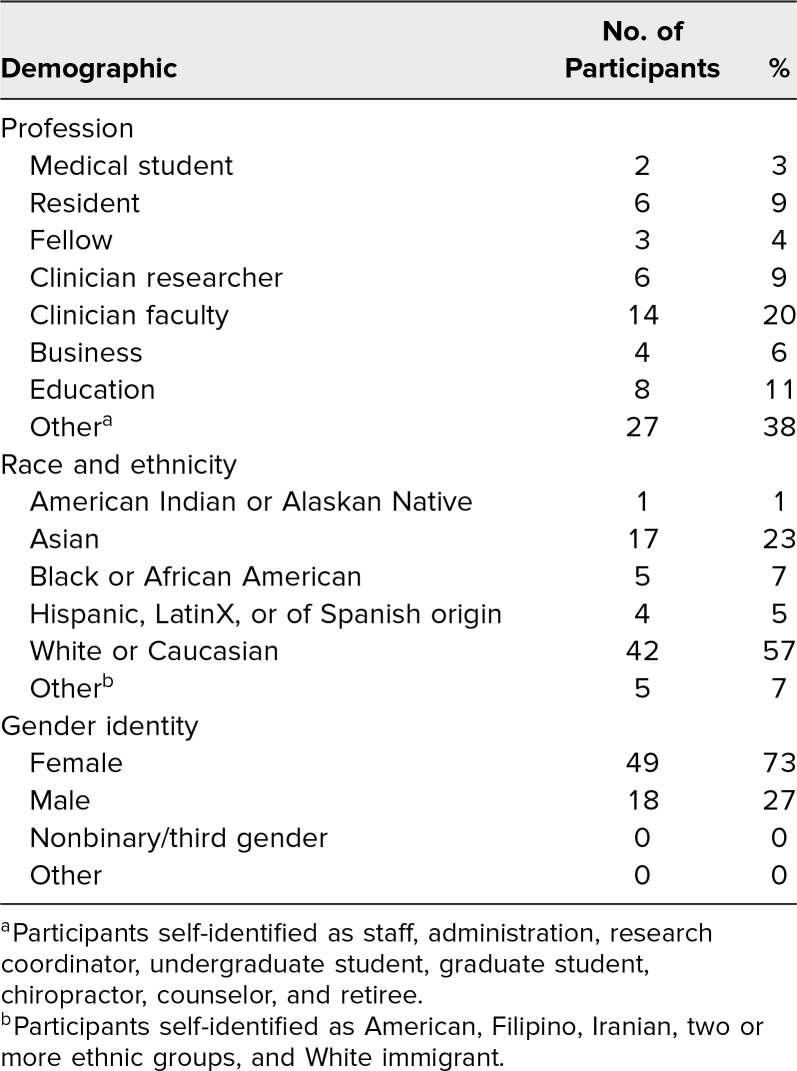
Participant Demographics

Participants felt that the workshop met learning objectives, with at least 90% somewhat agreeing or strongly agreeing with each individual objective (Table 2).

**Table 2.**
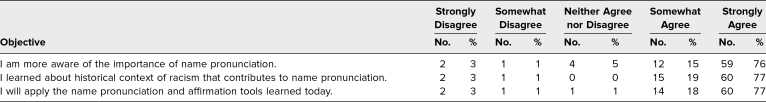
Postworkshop Participant Responses (*N* = 78) to Learning Objectives

Participants commented on personal and structural barriers to implementing lessons learned in the workshop. There were also several barriers related to other factors, including time, language, and hearing. When describing personal barriers, many participants reported feelings of discomfort or nervousness both when learning to correctly pronounce someone else's name and when correcting mispronunciation of their own. Participants also acknowledged structural barriers, with hierarchy or power dynamics preventing application of workshop lessons. Many participants reported no barriers to implementing workshop lessons (Table 3).

**Table 3.**
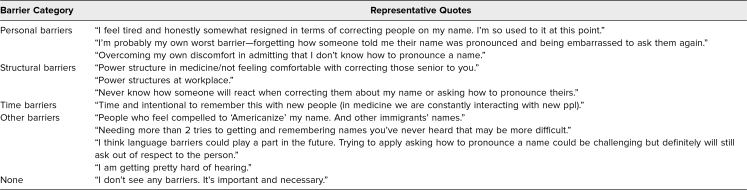
Participant Responses to Barriers to Implementation of Objectives

The most valued components of the workshop were the historical context, interactive small-group discussions, and resources for name pronunciation.

## Discussion

We created and delivered a workshop to teach the historical context of racism in chronic name mispronunciation, tools for correctly pronouncing names, and a framework for correcting name mispronunciation. Feedback from participants indicated that the workshop was well received and highlighted a topic that was seldom discussed or taught formally. Overall, postworkshop evaluations indicated that the workshop met learning objectives and that the structure and content were engaging, with small groups allowing for practical application of newly acquired skills.

Although there is literature in the educational and business sectors on the importance of names and effects of mispronunciation, there is a notable lack of such literature in medical education. Therefore, this workshop has been delivered primarily to health care professionals. However, it is designed to be generalizable to a wide variety of participants, although modifications may need to be made depending on participants in the group. For example, learners of different backgrounds may benefit from participating in small groups with learners of the same level to facilitate a safe discussion space.

This workshop provides an opportunity to engage in valuable discussion regarding personal narratives about names and their relationship to one's identity. While a majority of participants were White or Caucasian, participants were from diverse professional and racial and ethnic backgrounds, which added to the richness of small-group discussion. We have included examples of names from different backgrounds to offer exposure to a variety of names regardless of participant demographics. There are opportunities to include voice examples of names in different languages, address the distinction between names and pronouns, and discuss both professional and nonprofessional titles (e.g., Dr., Honor, Father, Mr., Ms. Mrs., etc.).

This workshop was presented in a virtual format due to the COVID-19 pandemic, but it would also be effective in person with some slight modifications, including space consideration for small groups and adaptation of some of the virtual exercises (e.g., the phonetic pronunciation virtual exercise could be converted to paper). The workshop has been presented at various medical and educational conferences, and it lends itself to a variety of conference formats.

### Limitations

There was a low response rate, with only 78 out of 147 total participants (53%) completing the postworkshop evaluation. Consequently, some perspectives may not have been captured. To address the low response rate, additional iterations of the workshop embedded the postworkshop survey prior to the final slides, rather than at the end, to allow time during the presentation and encourage participation.

To increase survey participation, only a single postworkshop survey was administered, rather than pre- and postworkshop surveys. This survey format has the potential to introduce recall bias, and pre- and postworkshop surveys would be beneficial for future workshops.

This workshop is a brief intervention and does not afford evidence of long-term knowledge or skills attainment or behavior modification. To assess long-term effects, it would be helpful to survey participants again at other intervals, such as 6 months and 1 year after the workshop.

Because the workshop was conducted virtually at conferences with other simultaneous workshops, participants had the option to enter and exit the workshop at any point. This made it difficult to assess the survey response rate, since it was challenging to track how long people were present.

### Conclusions

One's name is deeply tied to one's identity. Given that chronic name mispronunciation is a microaggression, it is critically important not only to be aware of the historical context but also to learn strategies for the correct pronunciation of names. Emphasis on correct name pronunciation provides a more equitable and inclusive environment for clinicians and patients alike.

## Appendices


Say My Name Presentation.pptxFacilitator Guide.docxParticipant Handout.docxPostworkshop Evaluation Form.docx
*All appendices are peer reviewed as integral parts of the Original Publication.*
