# Supplementary material for: Say My Name: Understanding the Power of Names, Correct Pronunciation, and Personal Narratives
Source: MedEdPORTAL. 2022 Nov 29;18:11284. doi: 10.15766/mep_2374-8265.11284 (PMC9705275; doi:10.15766/mep_2374-8265.11284)
Supplement: Supplementary file 1 — Say My Name Presentation.pptxFacilitator Guide.docxParticipant Handout.docxPostworkshop Evaluation Form.docx [file mep_2374-8265.11284-s001.zip › A. Say My Name Presentation.pptx]

## Slide 1
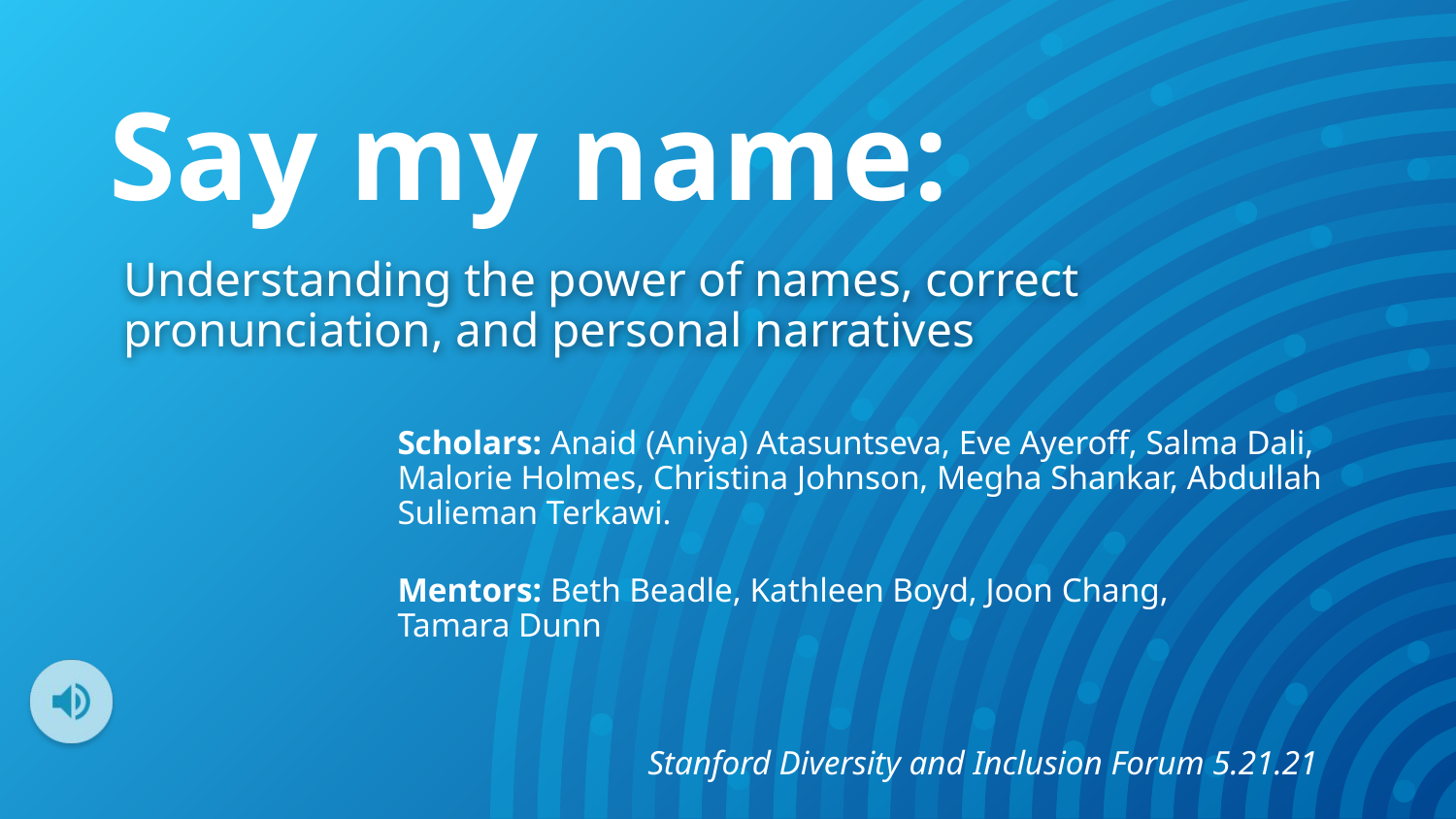

# Say my name:
Understanding the power of names, correct pronunciation, and personal narratives
Scholars: Anaid (Aniya) Atasuntseva, Eve Ayeroff, Salma Dali, Malorie Holmes, Christina Johnson, Megha Shankar, Abdullah Sulieman Terkawi.
Mentors: Beth Beadle, Kathleen Boyd, Joon Chang, Tamara Dunn
Stanford Diversity and Inclusion Forum 5.21.21

## Slide 2
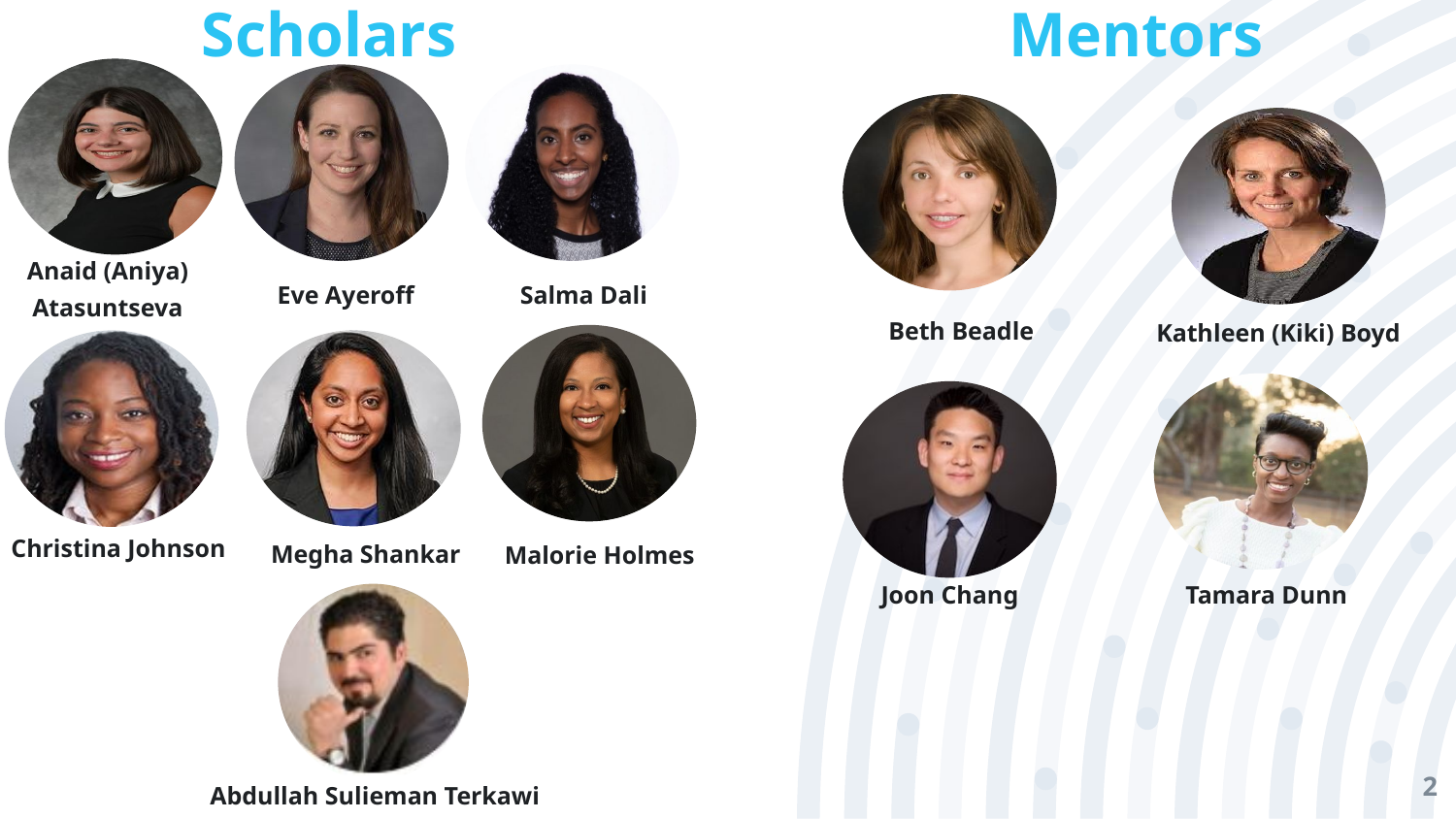

# Scholars
Mentors
Anaid (Aniya)
Atasuntseva
Eve Ayeroff
Salma Dali
Beth Beadle
Kathleen (Kiki) Boyd
Christina Johnson
Megha Shankar
Malorie Holmes
Joon Chang
Tamara Dunn
2
Abdullah Sulieman Terkawi

## Slide 3
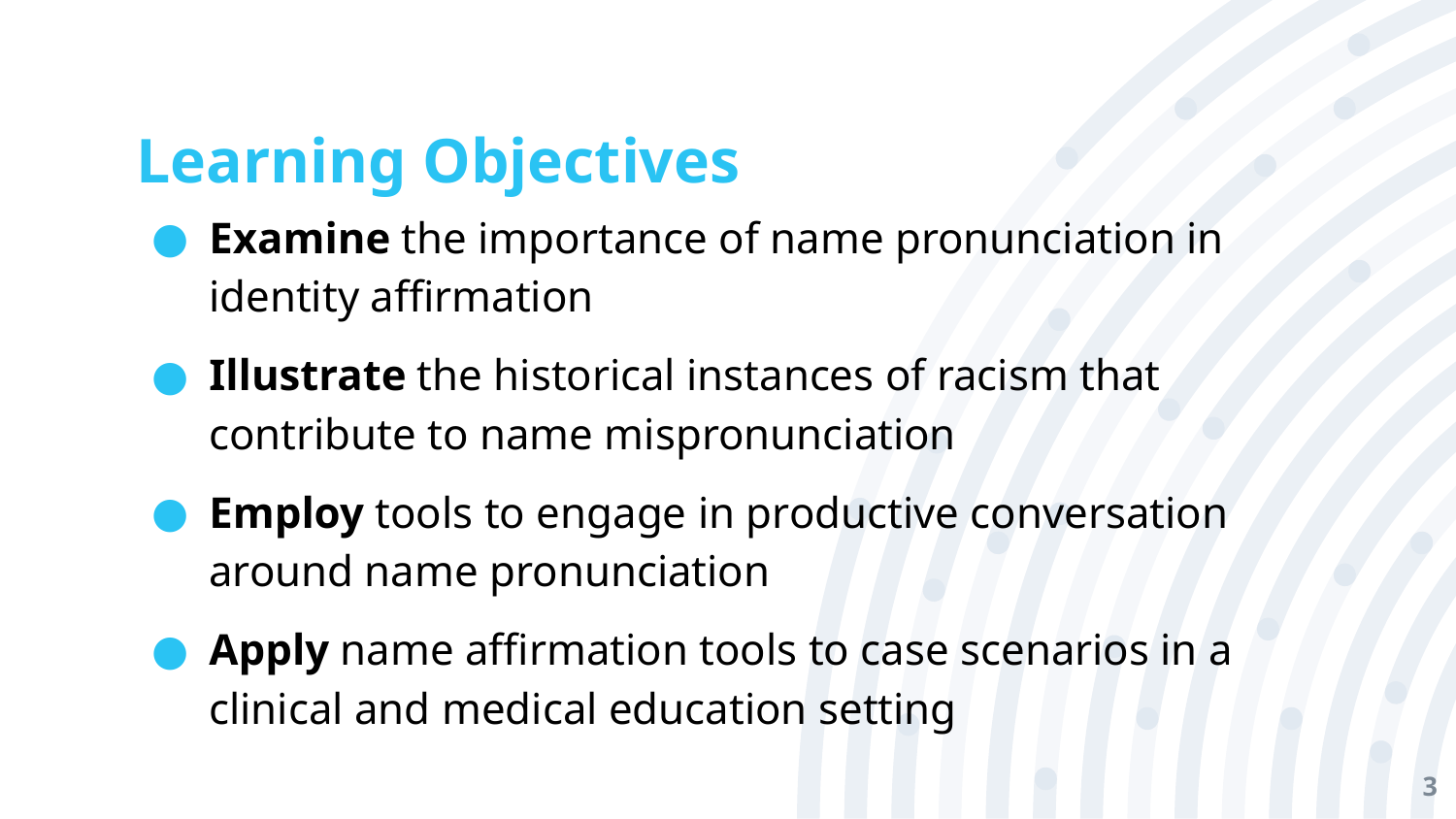

# Learning Objectives
Examine the importance of name pronunciation in identity affirmation
Illustrate the historical instances of racism that contribute to name mispronunciation
Employ tools to engage in productive conversation around name pronunciation
Apply name affirmation tools to case scenarios in a clinical and medical education setting
3

## Slide 4
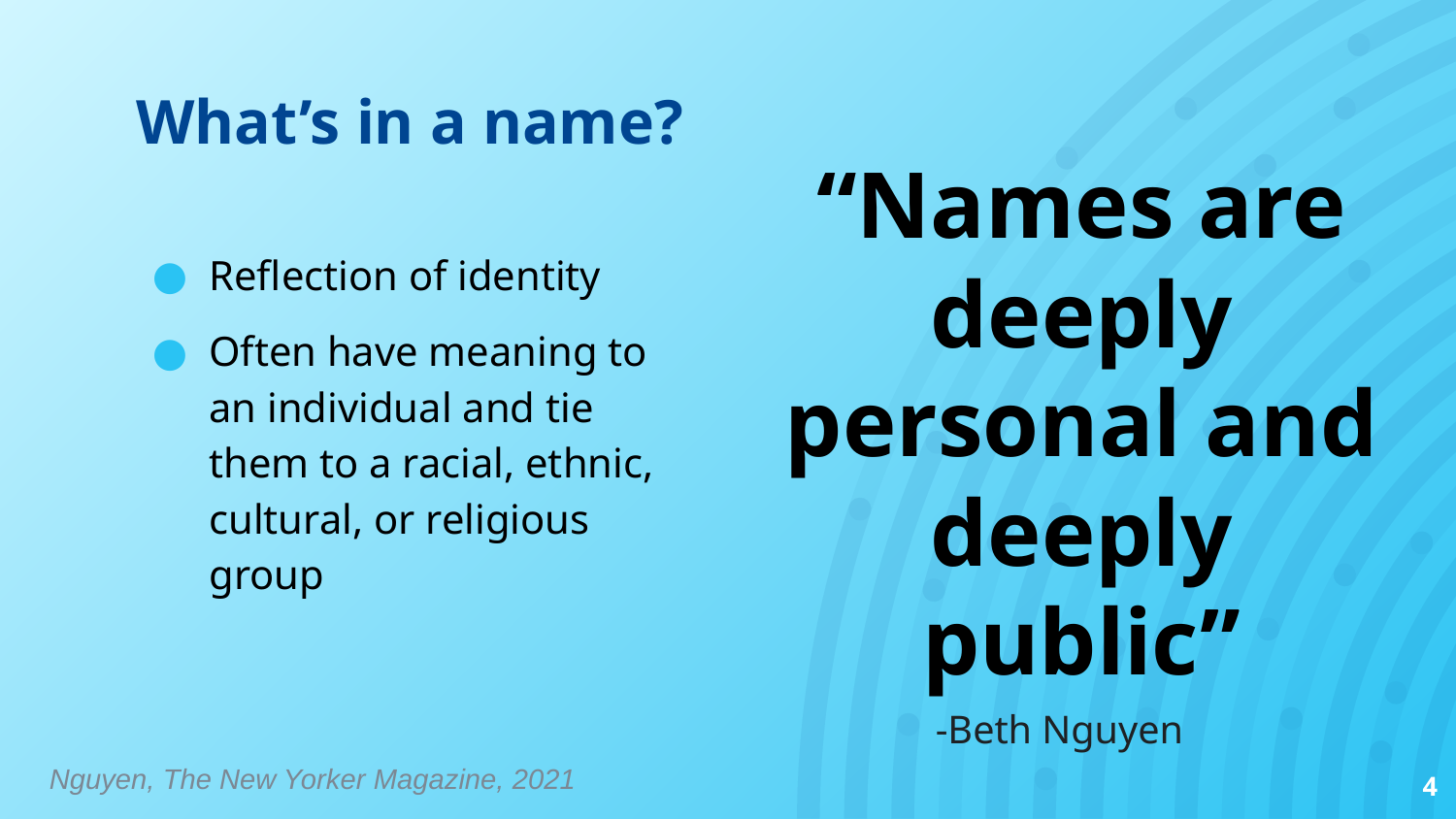

# What’s in a name?
“Names are deeply personal and deeply public”
Reflection of identity
Often have meaning to an individual and tie them to a racial, ethnic, cultural, or religious group
-Beth Nguyen
Nguyen, The New Yorker Magazine, 2021
4

## Slide 5
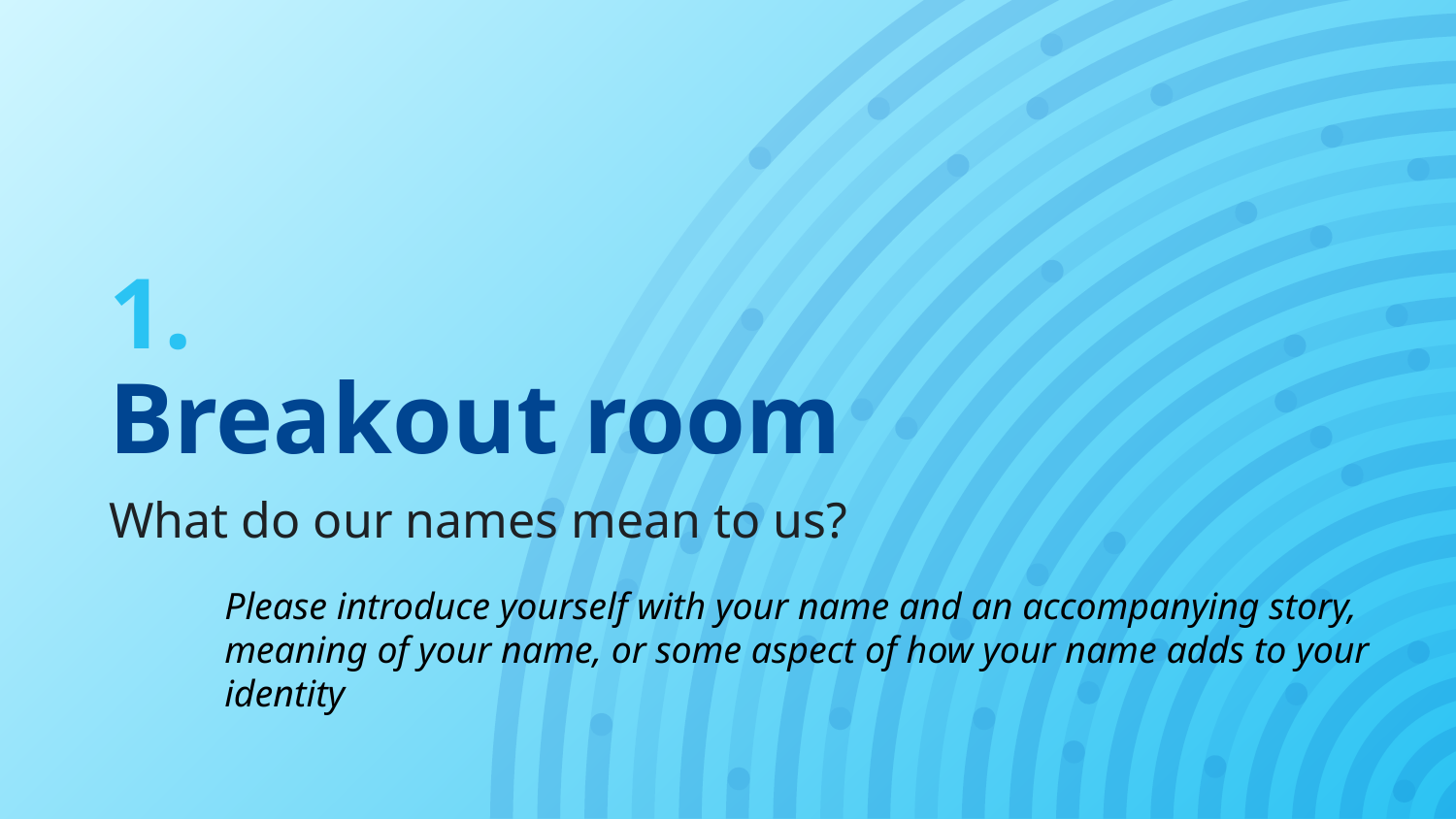

# 1.
Breakout room
What do our names mean to us?
Please introduce yourself with your name and an accompanying story, meaning of your name, or some aspect of how your name adds to your identity

## Slide 6
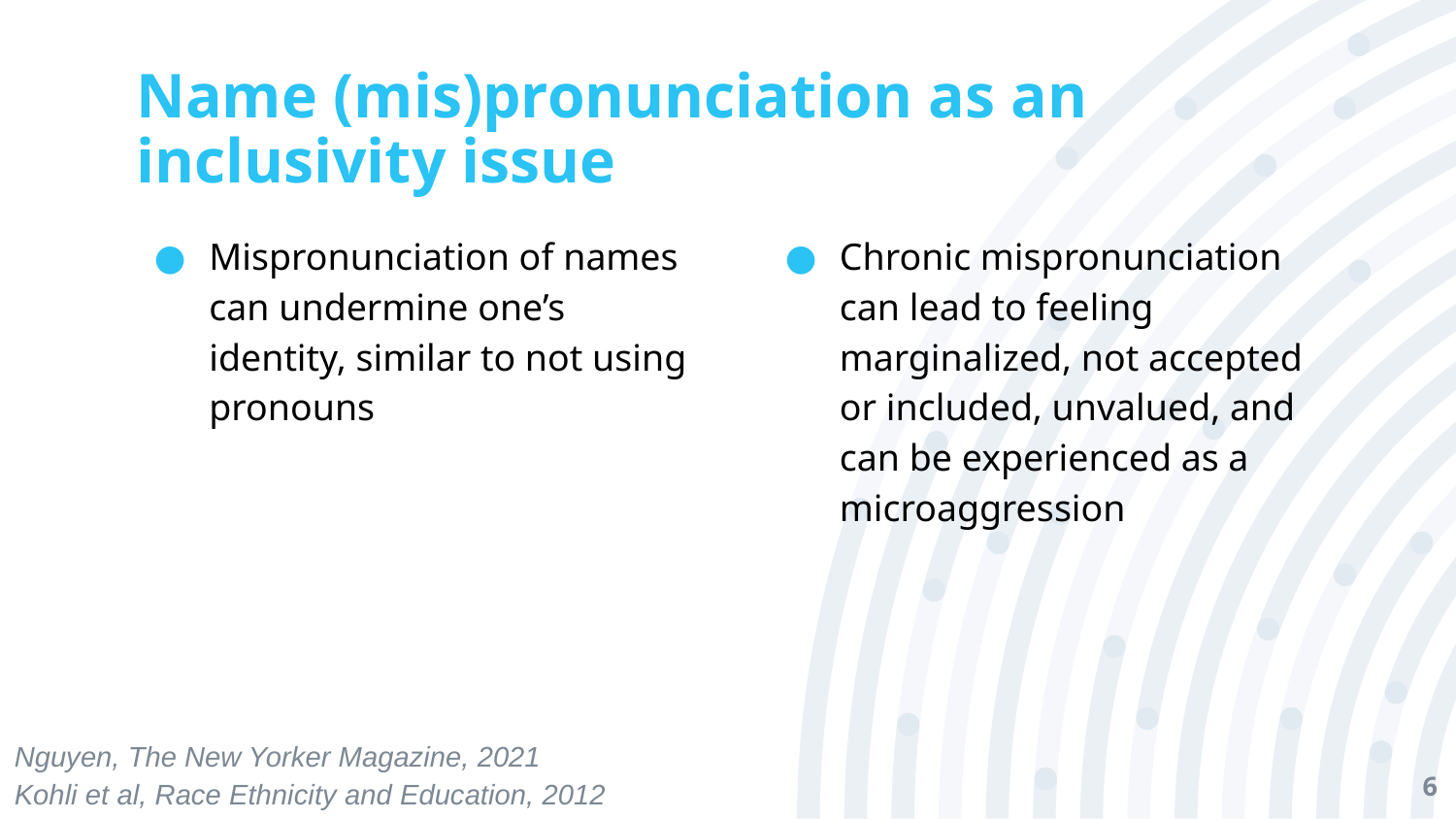

# Name (mis)pronunciation as an inclusivity issue
Mispronunciation of names can undermine one’s identity, similar to not using pronouns
Chronic mispronunciation can lead to feeling marginalized, not accepted or included, unvalued, and can be experienced as a microaggression
Nguyen, The New Yorker Magazine, 2021
6
Kohli et al, Race Ethnicity and Education, 2012

## Slide 7
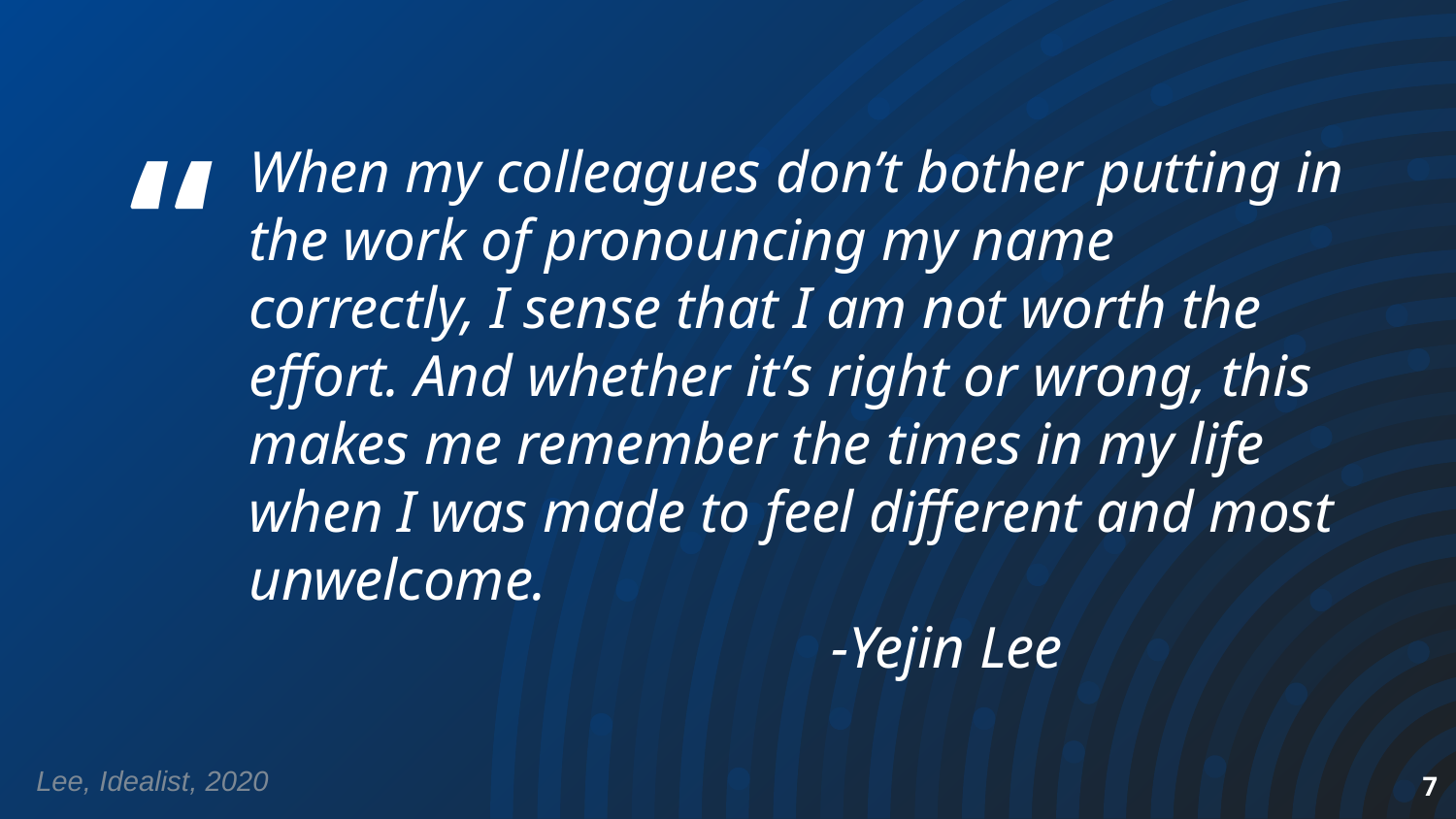

When my colleagues don’t bother putting in the work of pronouncing my name correctly, I sense that I am not worth the effort. And whether it’s right or wrong, this makes me remember the times in my life when I was made to feel different and most unwelcome.
			-Yejin Lee
Lee, Idealist, 2020
7

## Slide 8
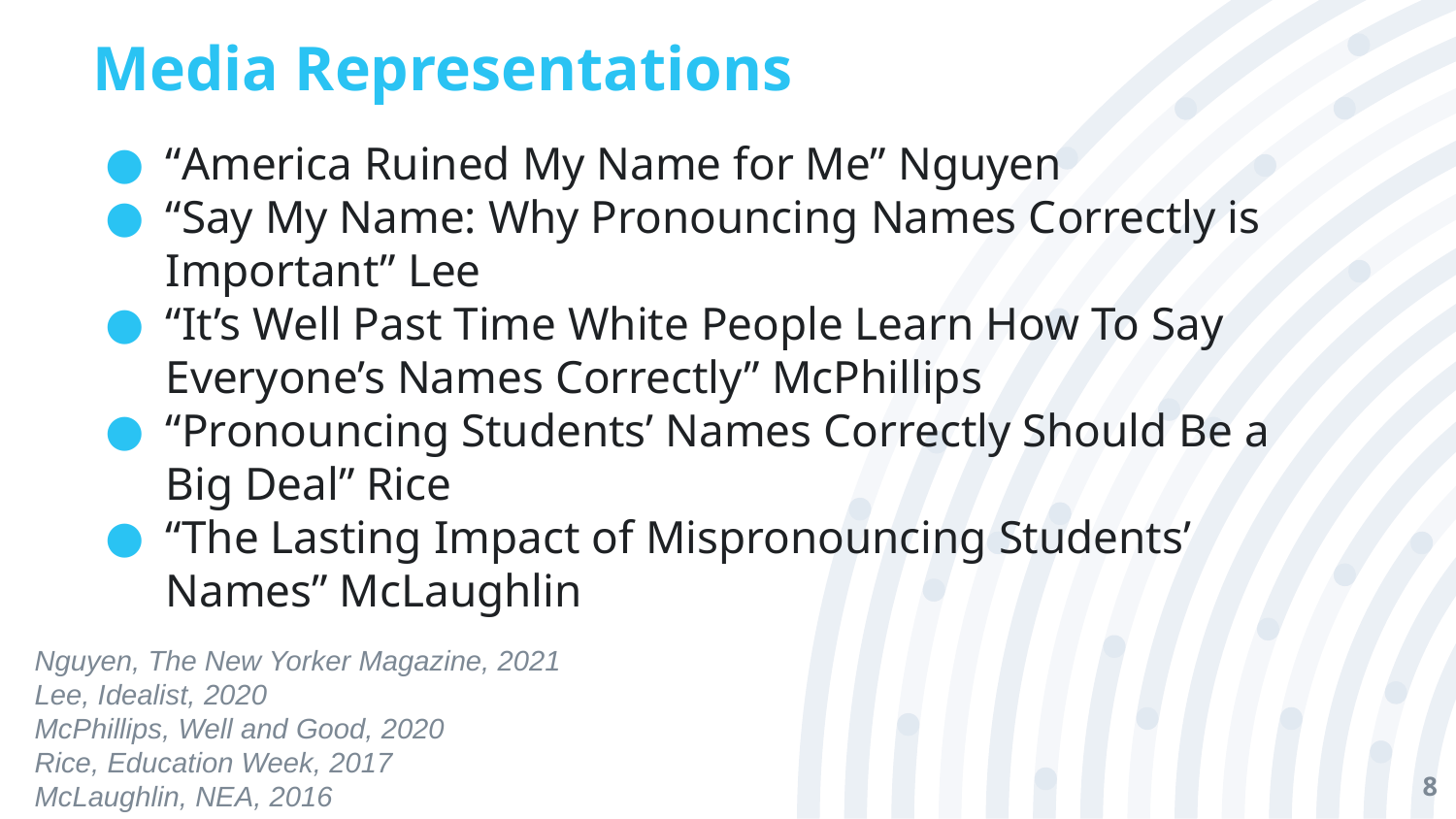

# Media Representations
“America Ruined My Name for Me” Nguyen
“Say My Name: Why Pronouncing Names Correctly is Important” Lee
“It’s Well Past Time White People Learn How To Say Everyone’s Names Correctly” McPhillips
“Pronouncing Students’ Names Correctly Should Be a Big Deal” Rice
“The Lasting Impact of Mispronouncing Students’ Names” McLaughlin
Nguyen, The New Yorker Magazine, 2021
Lee, Idealist, 2020
McPhillips, Well and Good, 2020
Rice, Education Week, 2017
McLaughlin, NEA, 2016
8

## Slide 9
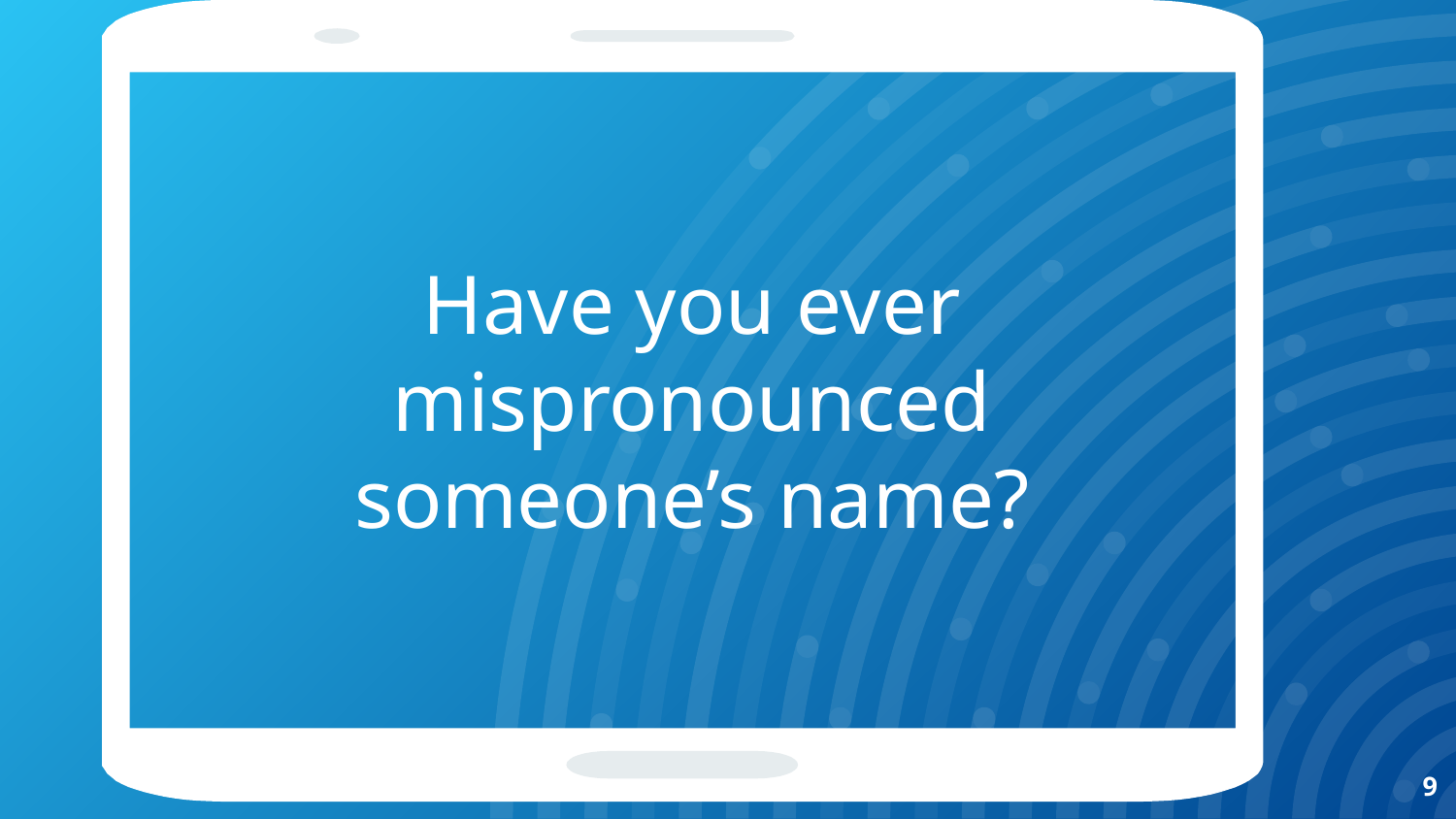

Have you ever mispronounced someone’s name?
9

## Slide 10
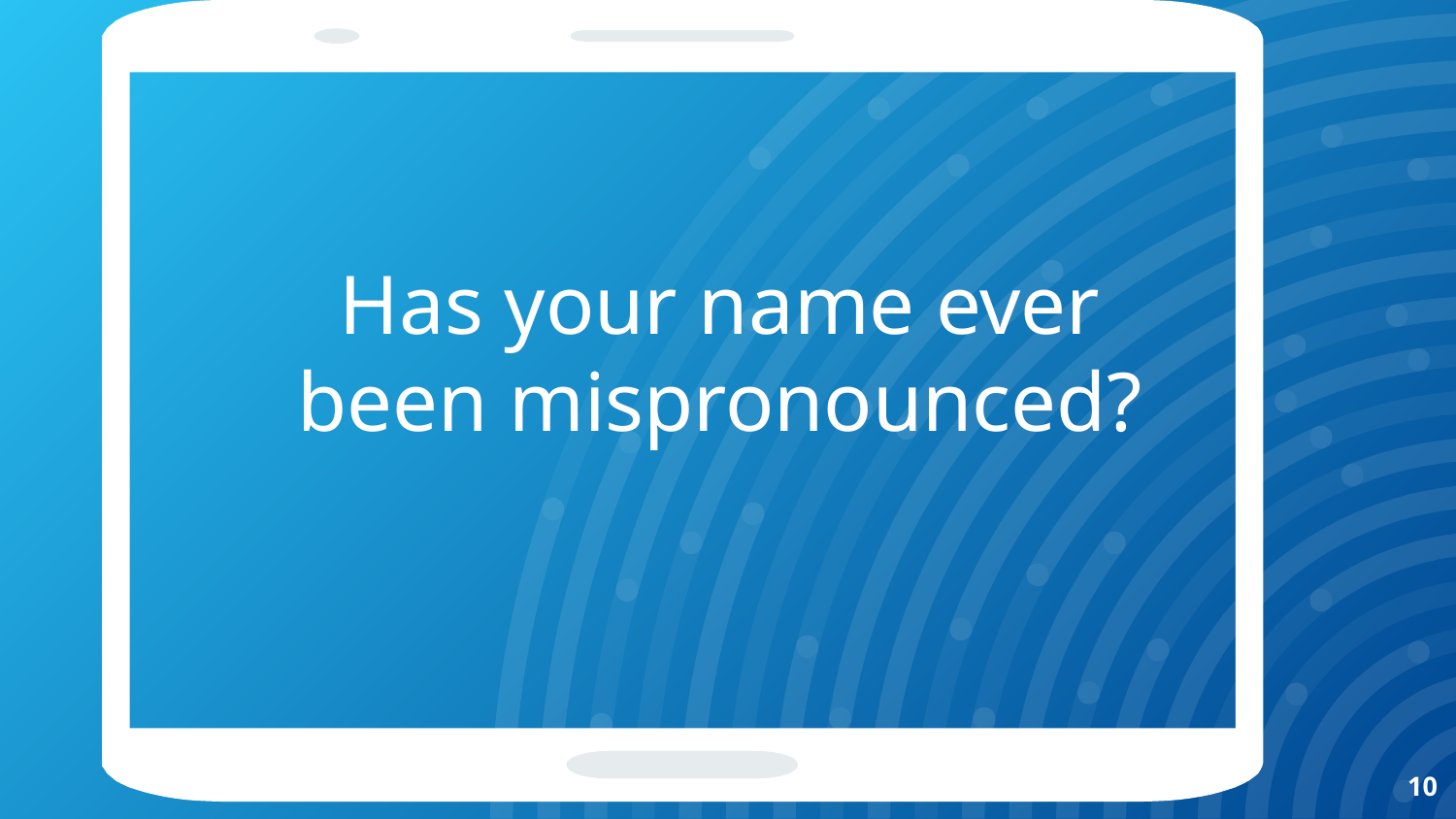

Has your name ever been mispronounced?
10

## Slide 11
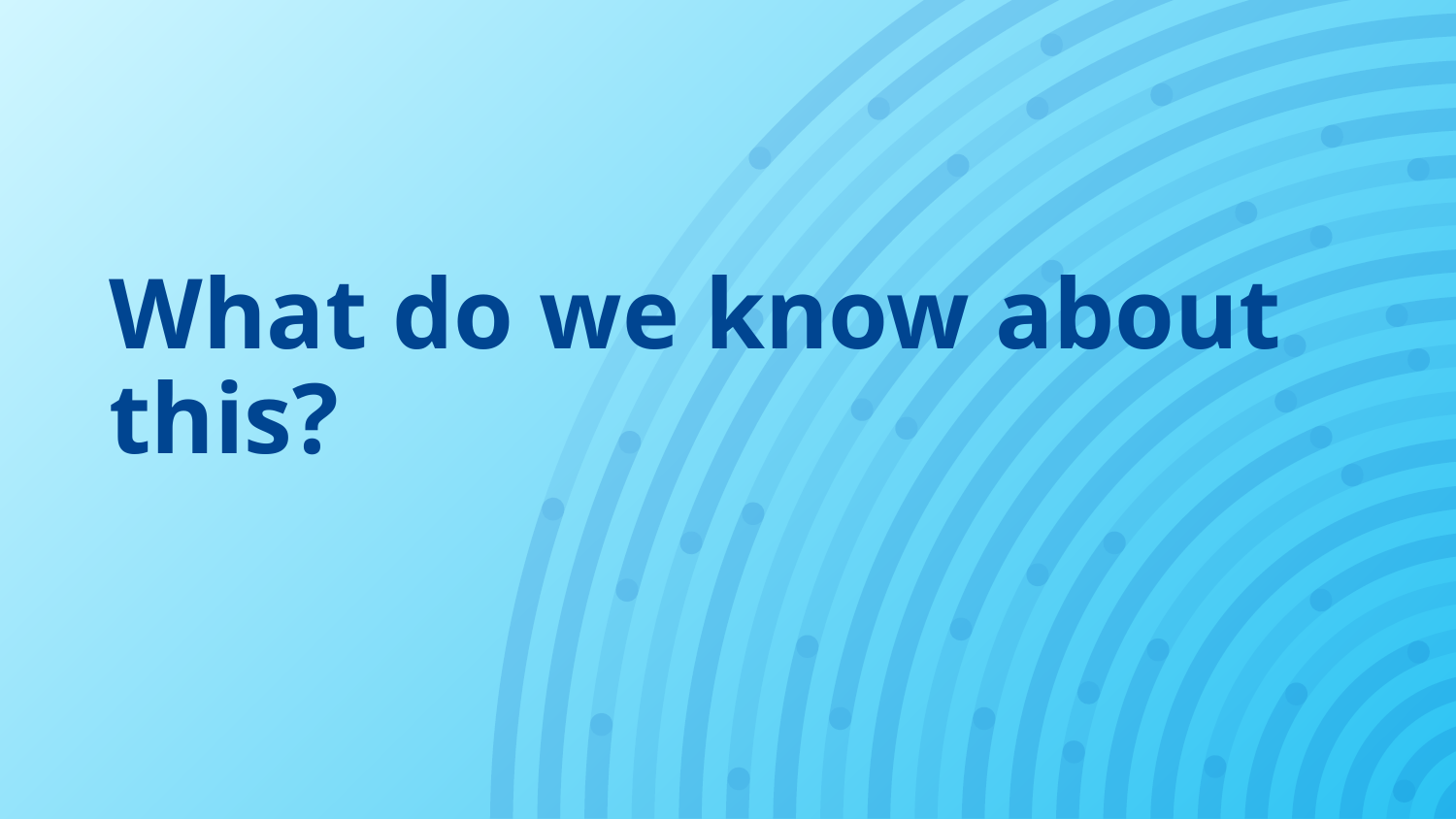

# What do we know about this?

## Slide 12
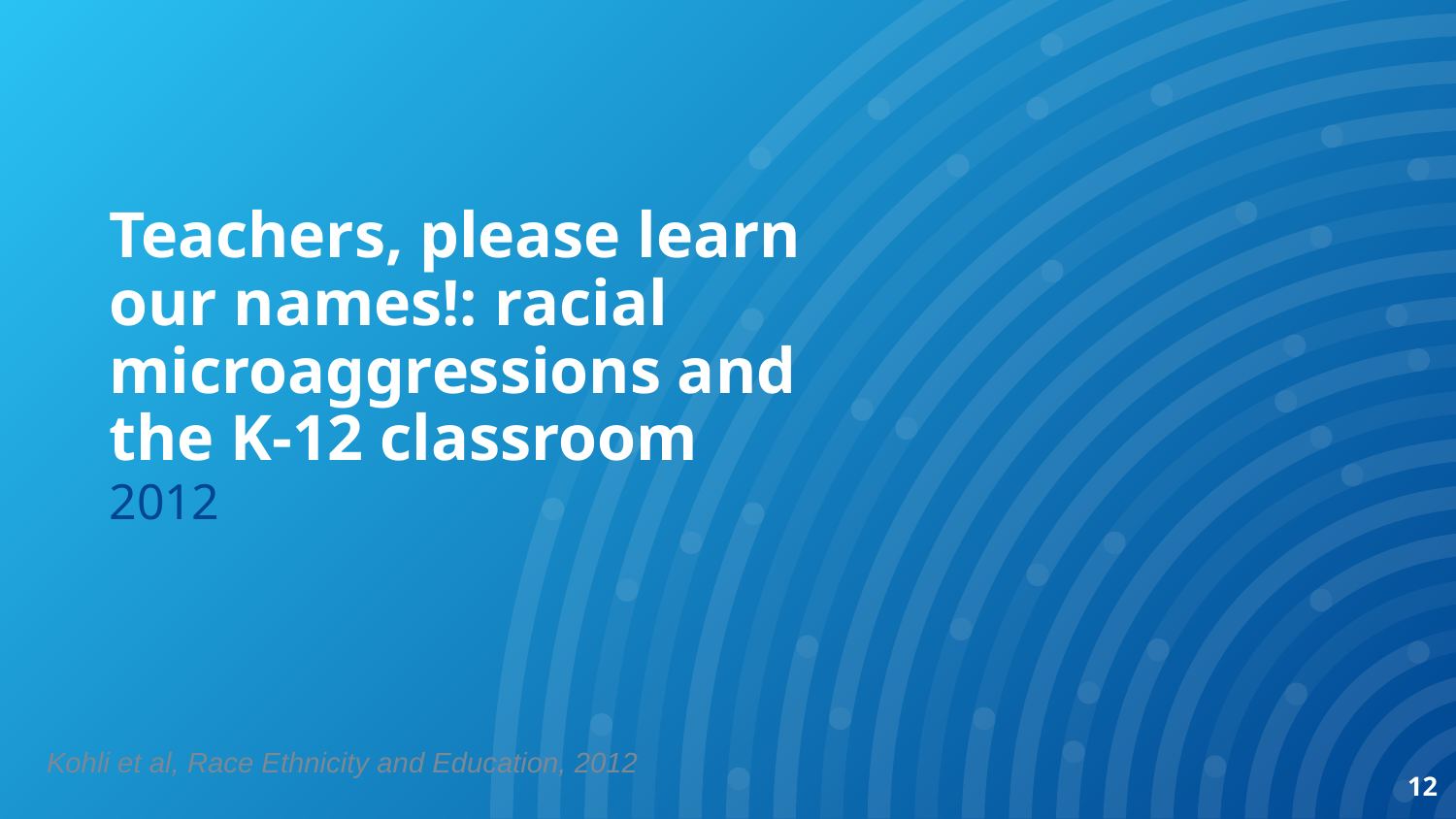

Teachers, please learn our names!: racial microaggressions and the K-12 classroom
2012
Kohli et al, Race Ethnicity and Education, 2012
12

## Slide 13
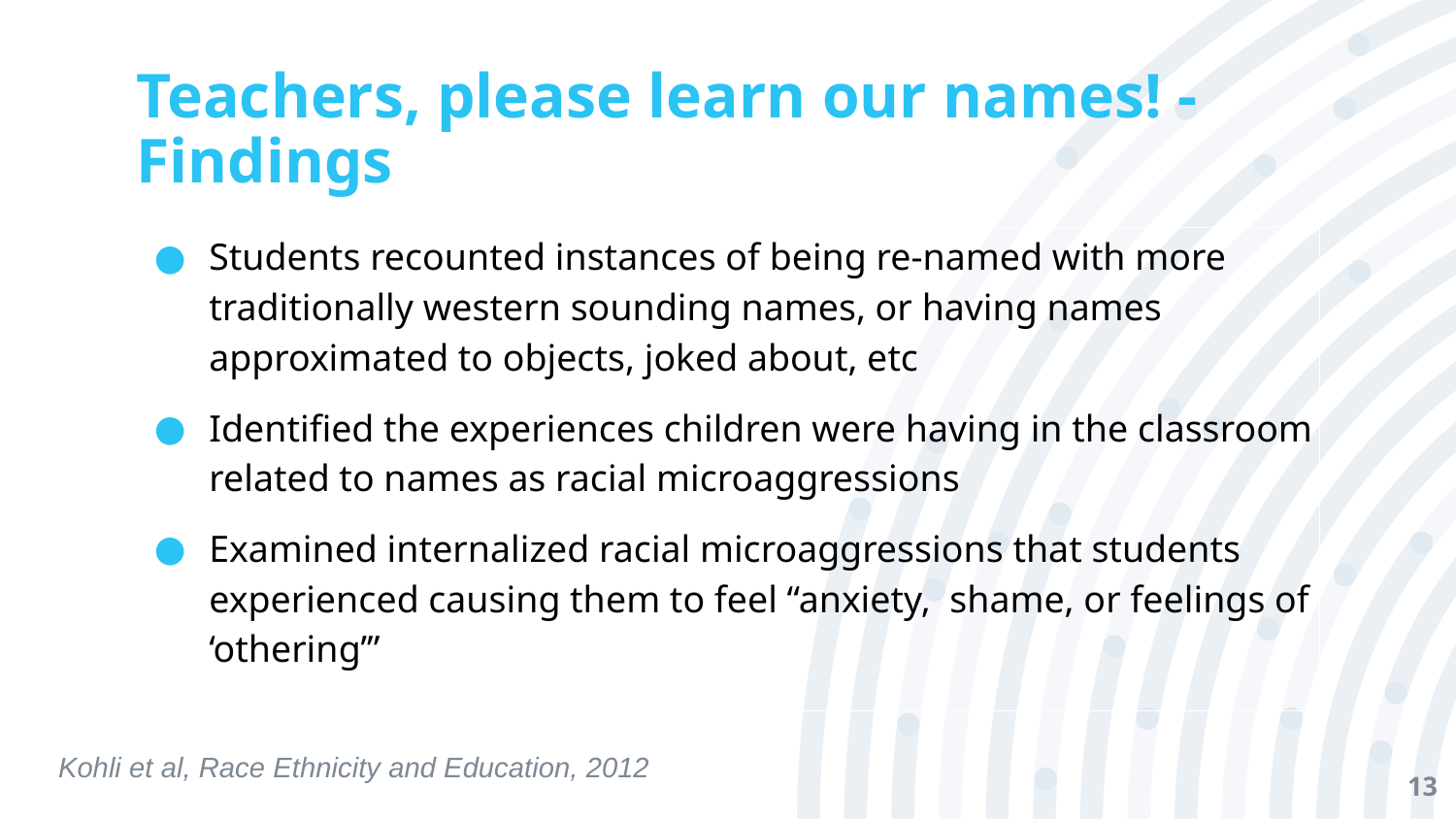

# Teachers, please learn our names! - Findings
Students recounted instances of being re-named with more traditionally western sounding names, or having names approximated to objects, joked about, etc
Identified the experiences children were having in the classroom related to names as racial microaggressions
Examined internalized racial microaggressions that students experienced causing them to feel “anxiety, shame, or feelings of ‘othering’”
Kohli et al, Race Ethnicity and Education, 2012
13

## Slide 14
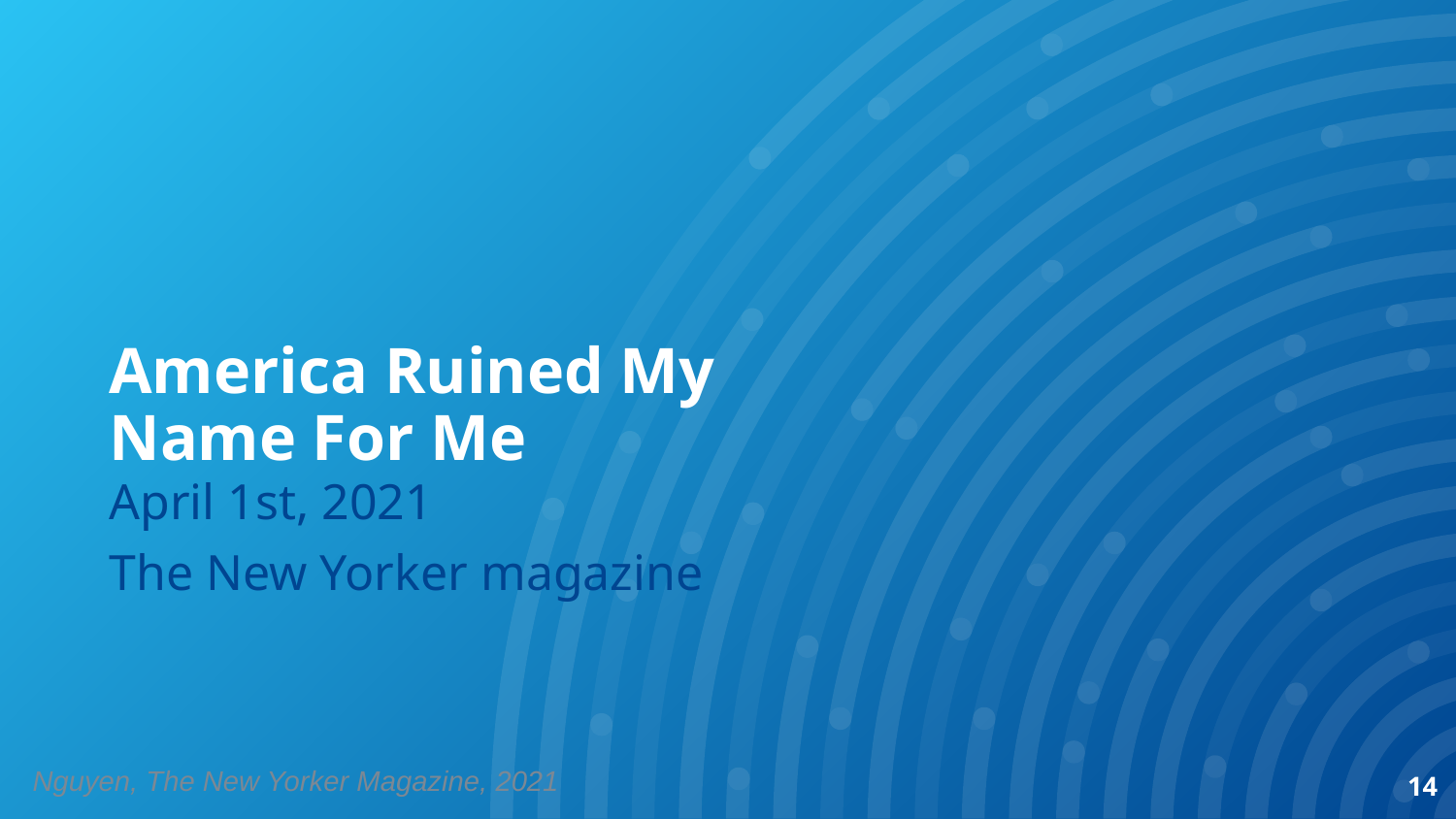

America Ruined My Name For Me
April 1st, 2021
The New Yorker magazine
Nguyen, The New Yorker Magazine, 2021
14

## Slide 15
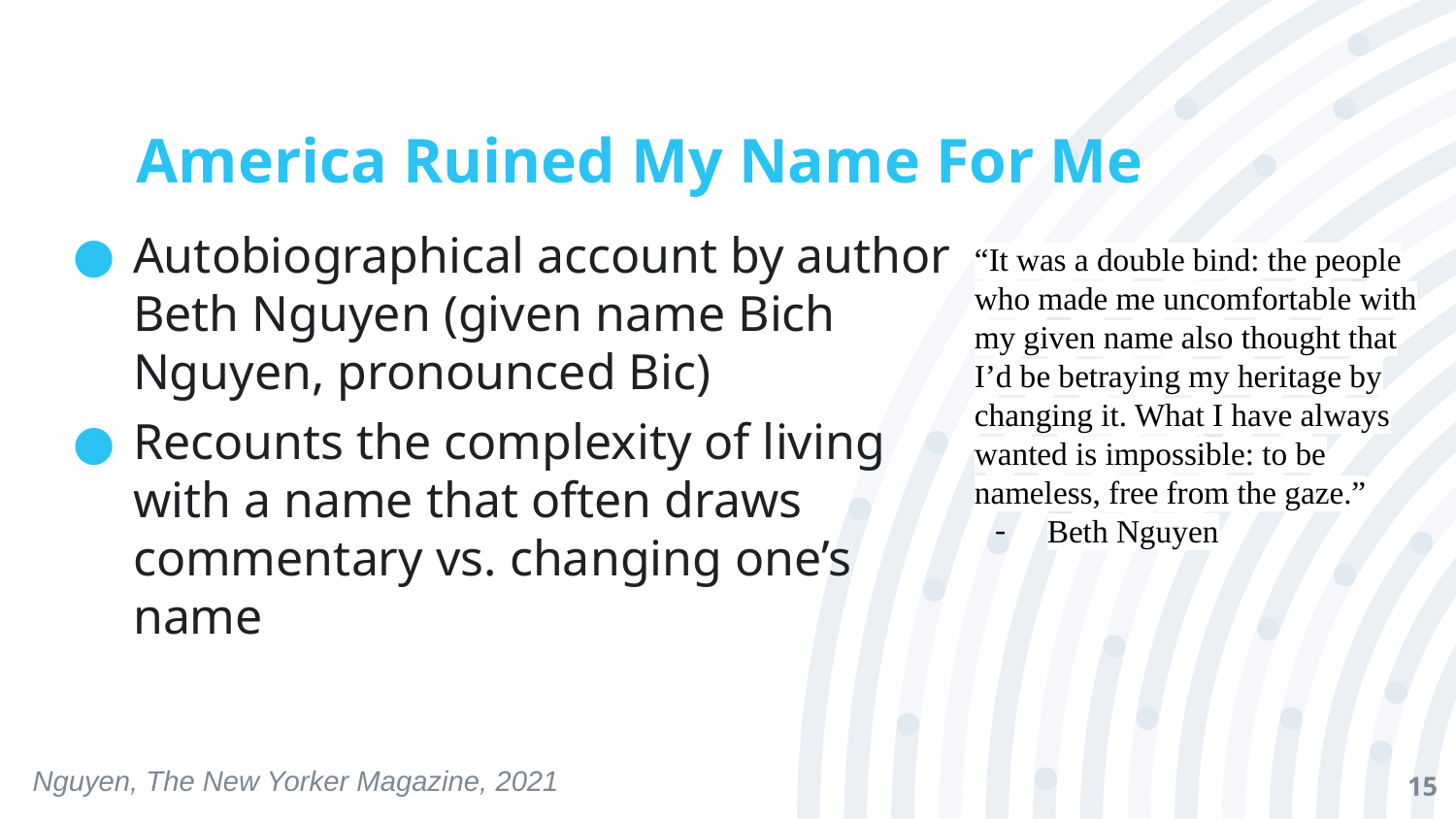

# America Ruined My Name For Me
Autobiographical account by author Beth Nguyen (given name Bich Nguyen, pronounced Bic)
Recounts the complexity of living with a name that often draws commentary vs. changing one’s name
“It was a double bind: the people who made me uncomfortable with my given name also thought that I’d be betraying my heritage by changing it. What I have always wanted is impossible: to be nameless, free from the gaze.”
Beth Nguyen
Nguyen, The New Yorker Magazine, 2021
15

## Slide 16
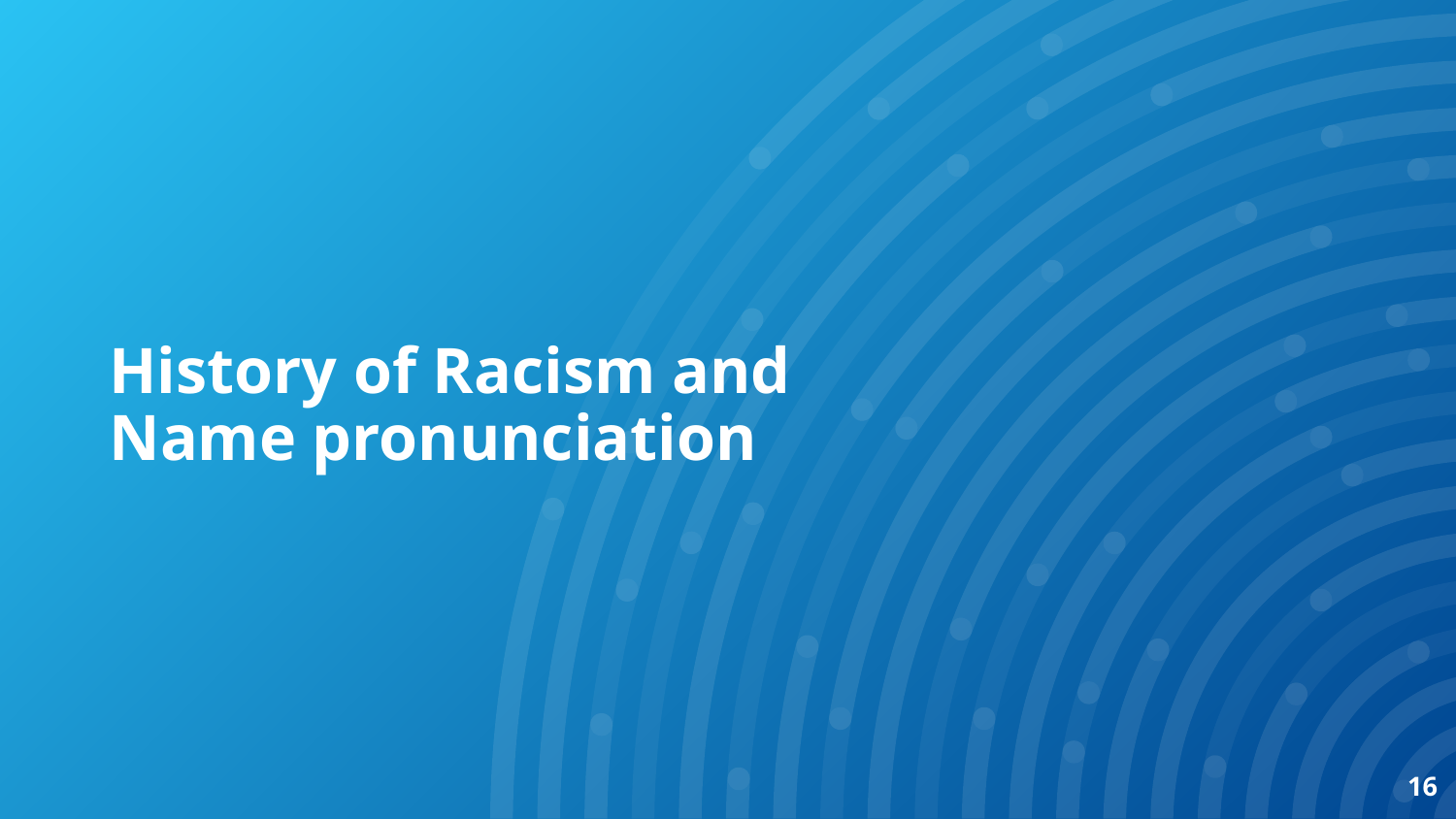

History of Racism and Name pronunciation
16

## Slide 17
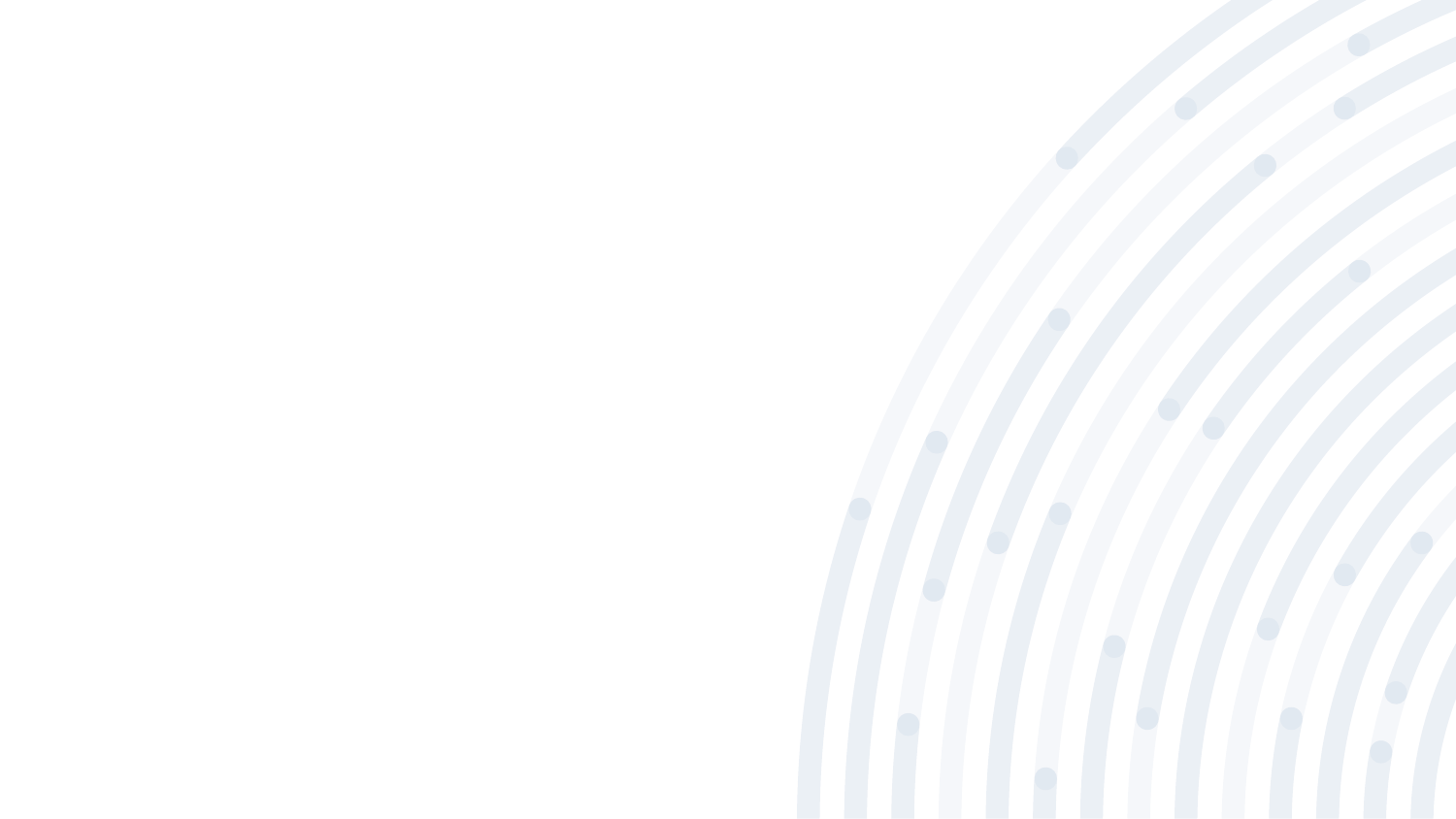

## Slide 18
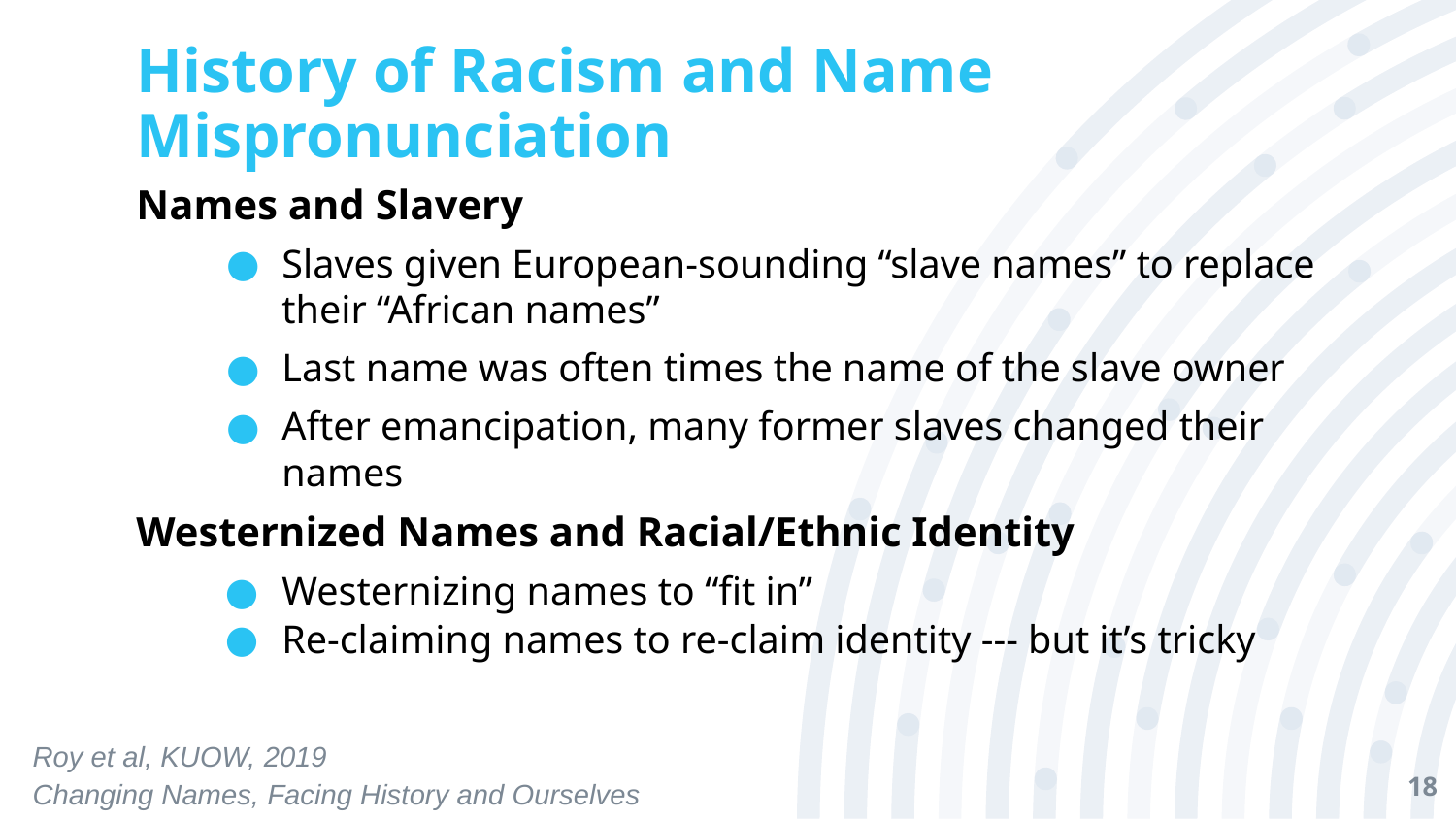

# History of Racism and Name Mispronunciation
Names and Slavery
Slaves given European-sounding “slave names” to replace their “African names”
Last name was often times the name of the slave owner
After emancipation, many former slaves changed their names
Westernized Names and Racial/Ethnic Identity
Westernizing names to “fit in”
Re-claiming names to re-claim identity --- but it’s tricky
Roy et al, KUOW, 2019
18
Changing Names, Facing History and Ourselves

## Slide 19
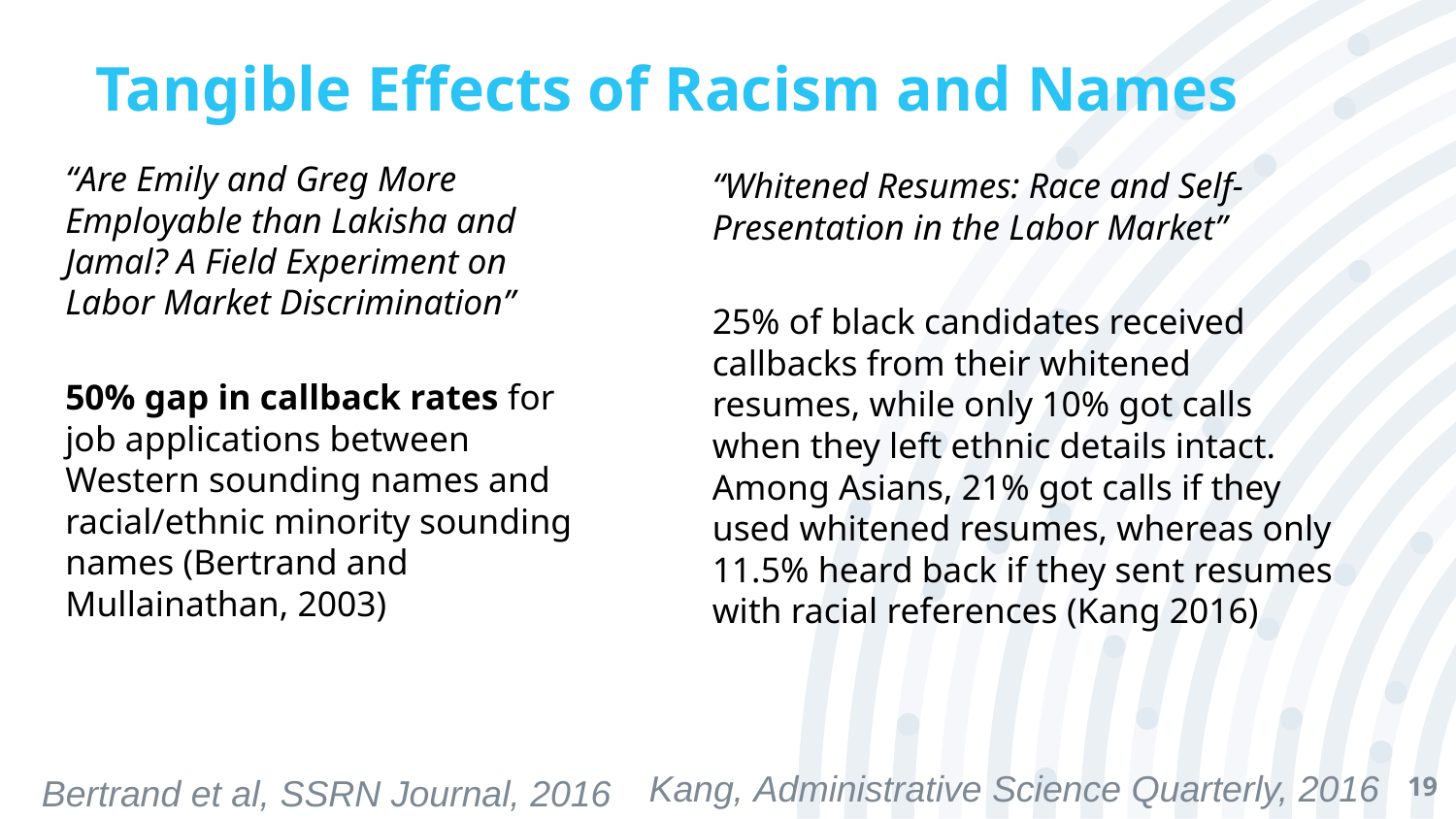

# Tangible Effects of Racism and Names
“Are Emily and Greg More Employable than Lakisha and Jamal? A Field Experiment on Labor Market Discrimination”
50% gap in callback rates for job applications between Western sounding names and racial/ethnic minority sounding names (Bertrand and Mullainathan, 2003)
“Whitened Resumes: Race and Self-Presentation in the Labor Market”
25% of black candidates received callbacks from their whitened resumes, while only 10% got calls when they left ethnic details intact. Among Asians, 21% got calls if they used whitened resumes, whereas only 11.5% heard back if they sent resumes with racial references (Kang 2016)
Kang, Administrative Science Quarterly, 2016
Bertrand et al, SSRN Journal, 2016
19

## Slide 20
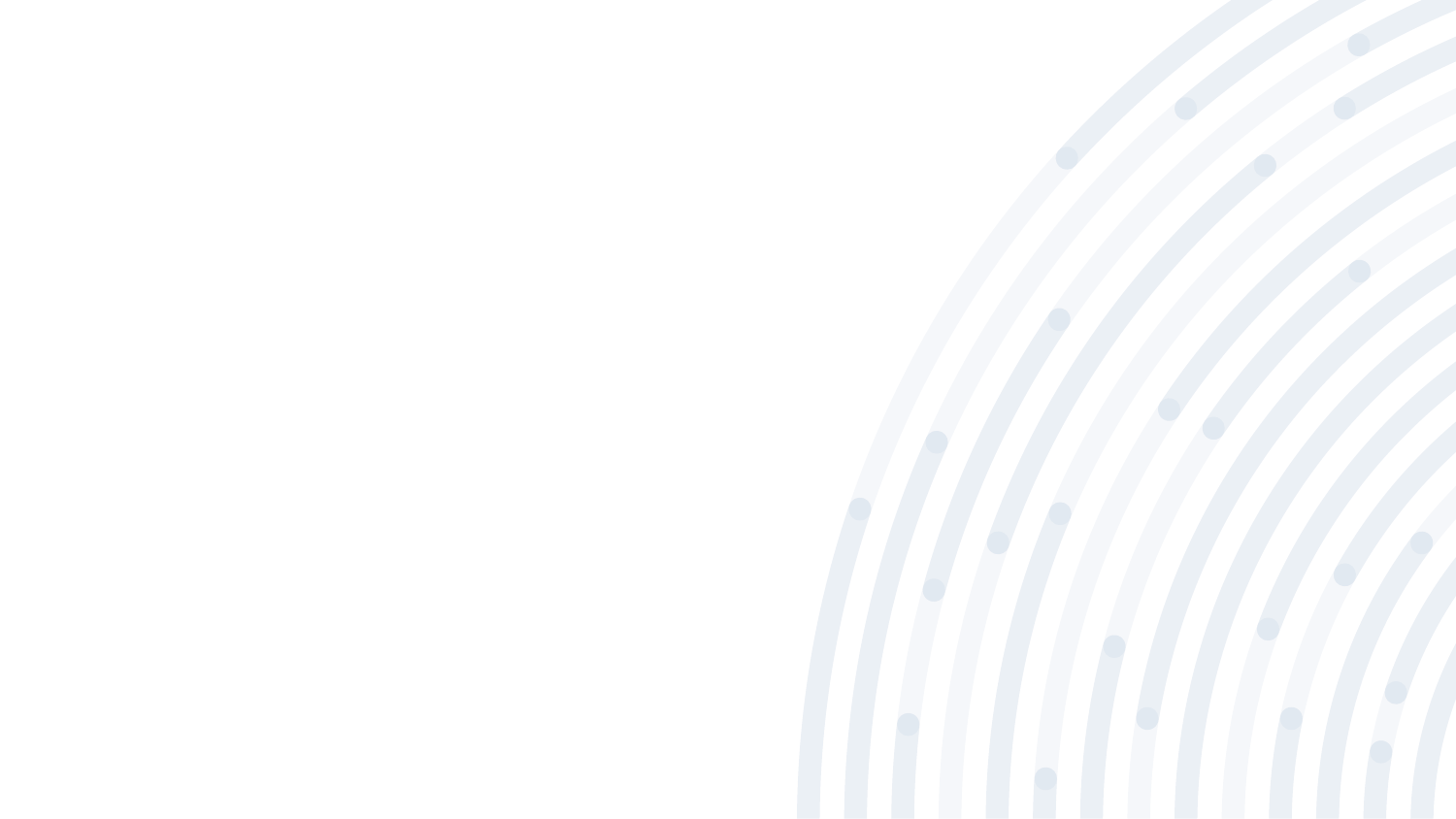

## Slide 21
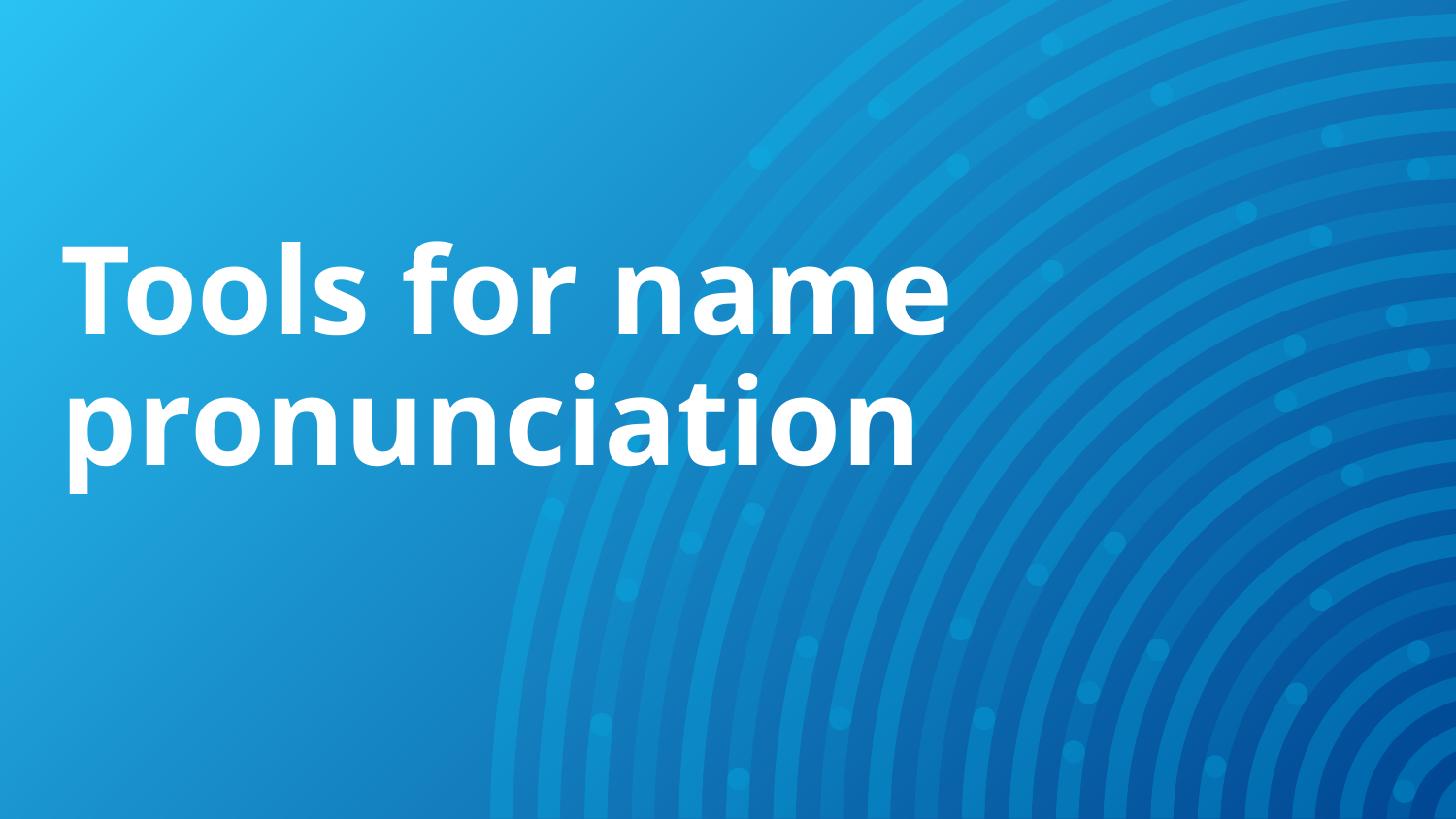

# Tools for name pronunciation

## Slide 22
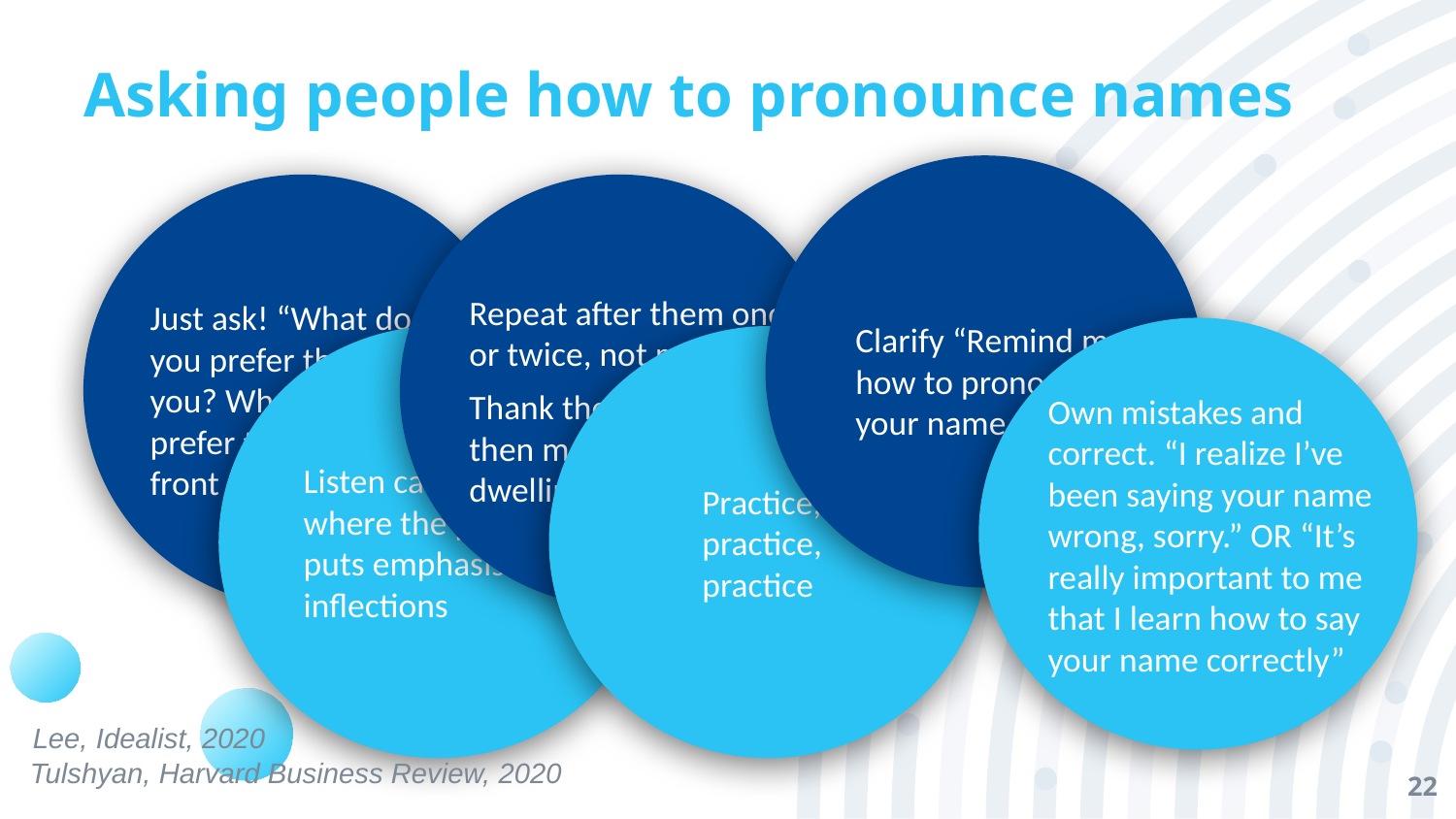

# Asking people how to pronounce names
Clarify “Remind me how to pronounce your name again”
Just ask! “What do you prefer that I call you? What do you prefer to be called in front of others?”
Repeat after them once or twice, not more
Thank them for sharing then move on, avoid dwelling
Own mistakes and correct. “I realize I’ve been saying your name wrong, sorry.” OR “It’s really important to me that I learn how to say your name correctly”
Listen carefully to where the person puts emphasis and inflections
Practice, practice, practice
Lee, Idealist, 2020
Tulshyan, Harvard Business Review, 2020
22

## Slide 23
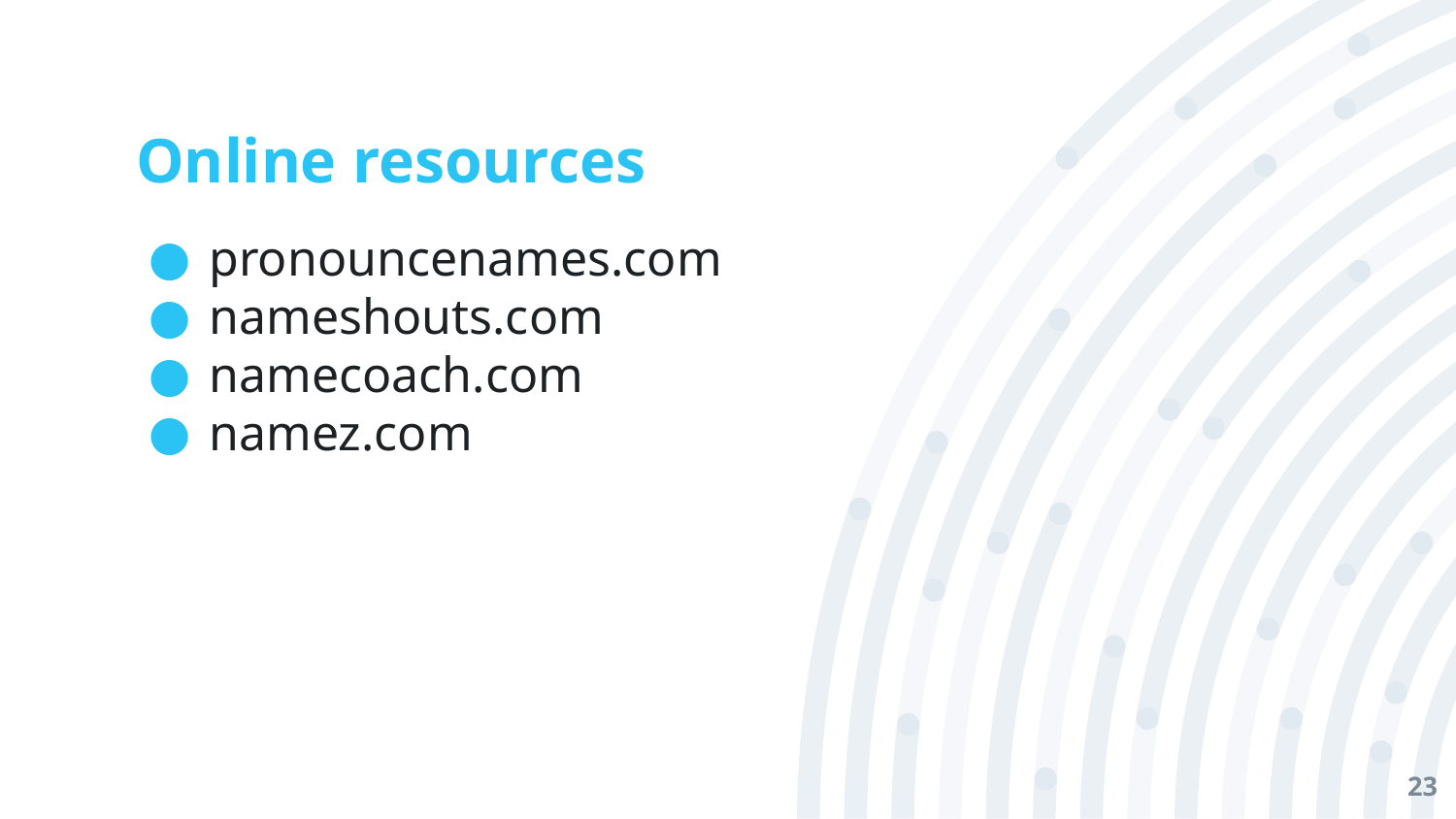

# Online resources
pronouncenames.com
nameshouts.com
namecoach.com
namez.com
23

## Slide 24
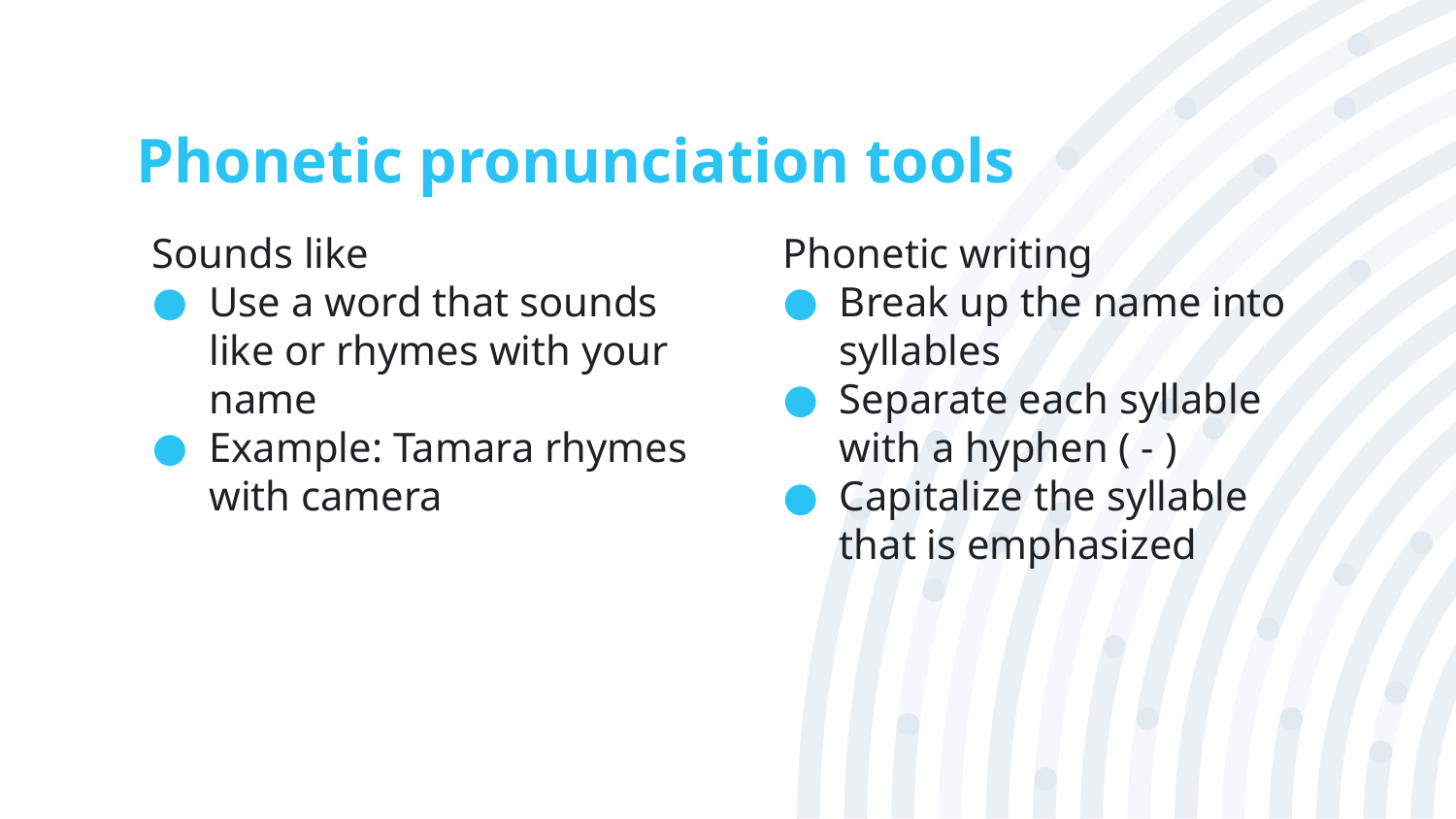

# Phonetic pronunciation tools
Sounds like
Use a word that sounds like or rhymes with your name
Example: Tamara rhymes with camera
Phonetic writing
Break up the name into syllables
Separate each syllable with a hyphen ( - )
Capitalize the syllable that is emphasized

## Slide 25
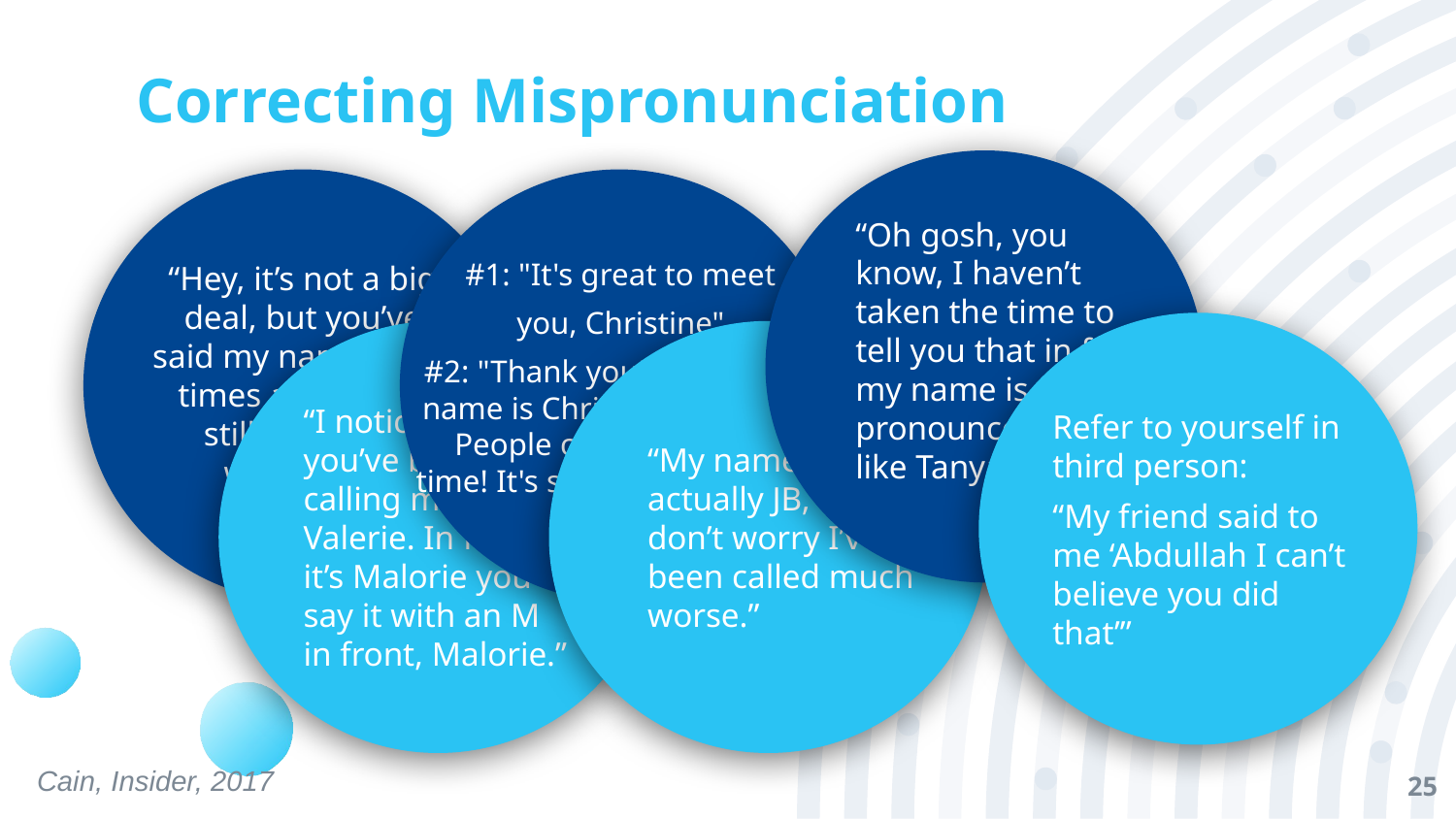

# Correcting Mispronunciation
“Oh gosh, you know, I haven’t taken the time to tell you that in fact my name is pronounced Aniya, like Tanya”
“Hey, it’s not a big deal, but you’ve said my name three times and you’re still getting it wrong. It’s actually...”
#1: "It's great to meet
you, Christine"
#2: "Thank you! Actually, my name is Christina with an 'a.' People confuse it all the time! It's so nice meeting you as well!"
Refer to yourself in third person:
“My friend said to me ‘Abdullah I can’t believe you did that’”
“I noticed that you’ve been calling me Valerie. In fact, it’s Malorie you say it with an M in front, Malorie.”
“My name is actually JB, but don’t worry I’ve been called much worse.”
Cain, Insider, 2017
25

## Slide 26
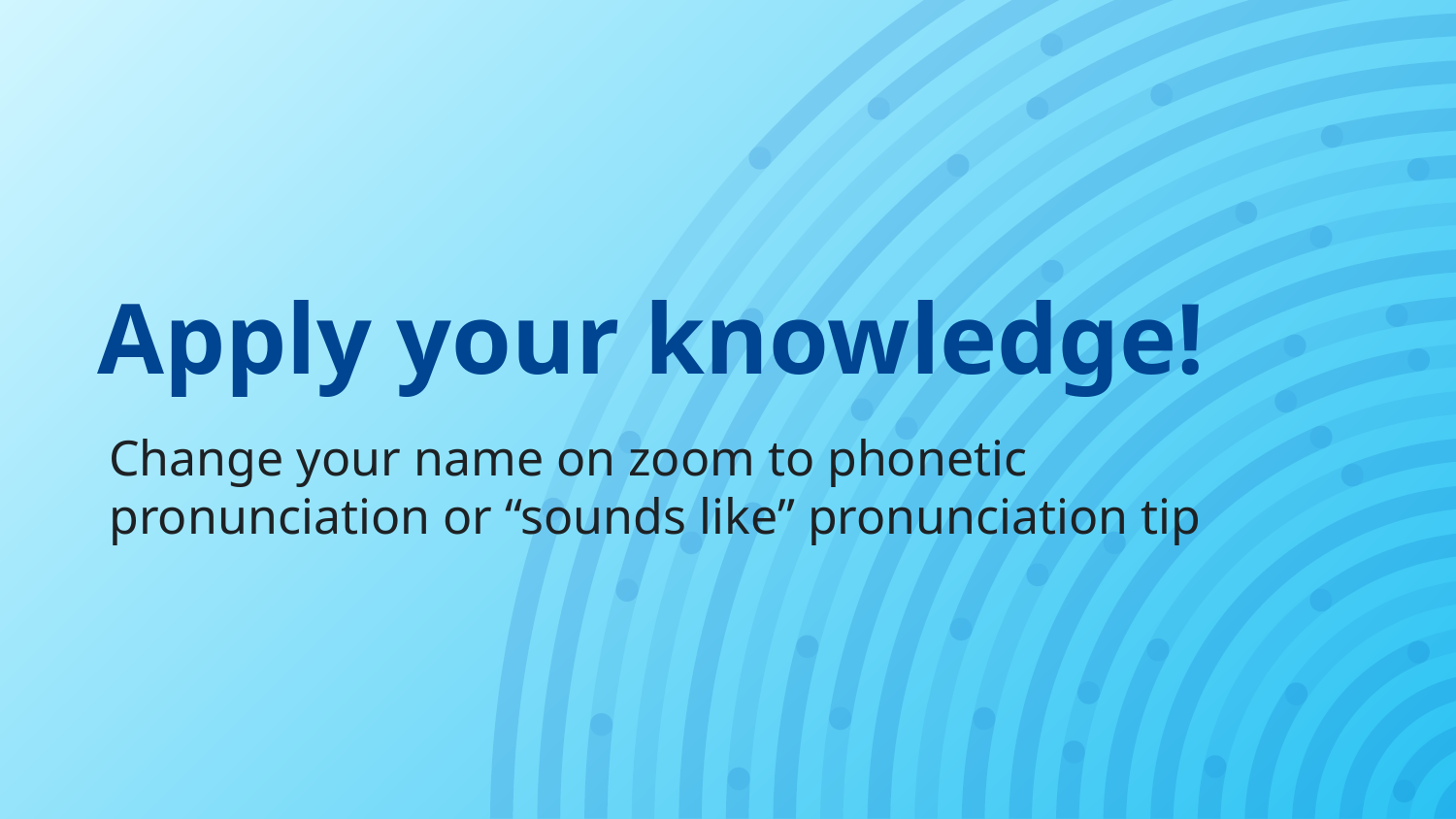

# Apply your knowledge!
Change your name on zoom to phonetic pronunciation or “sounds like” pronunciation tip

## Slide 27
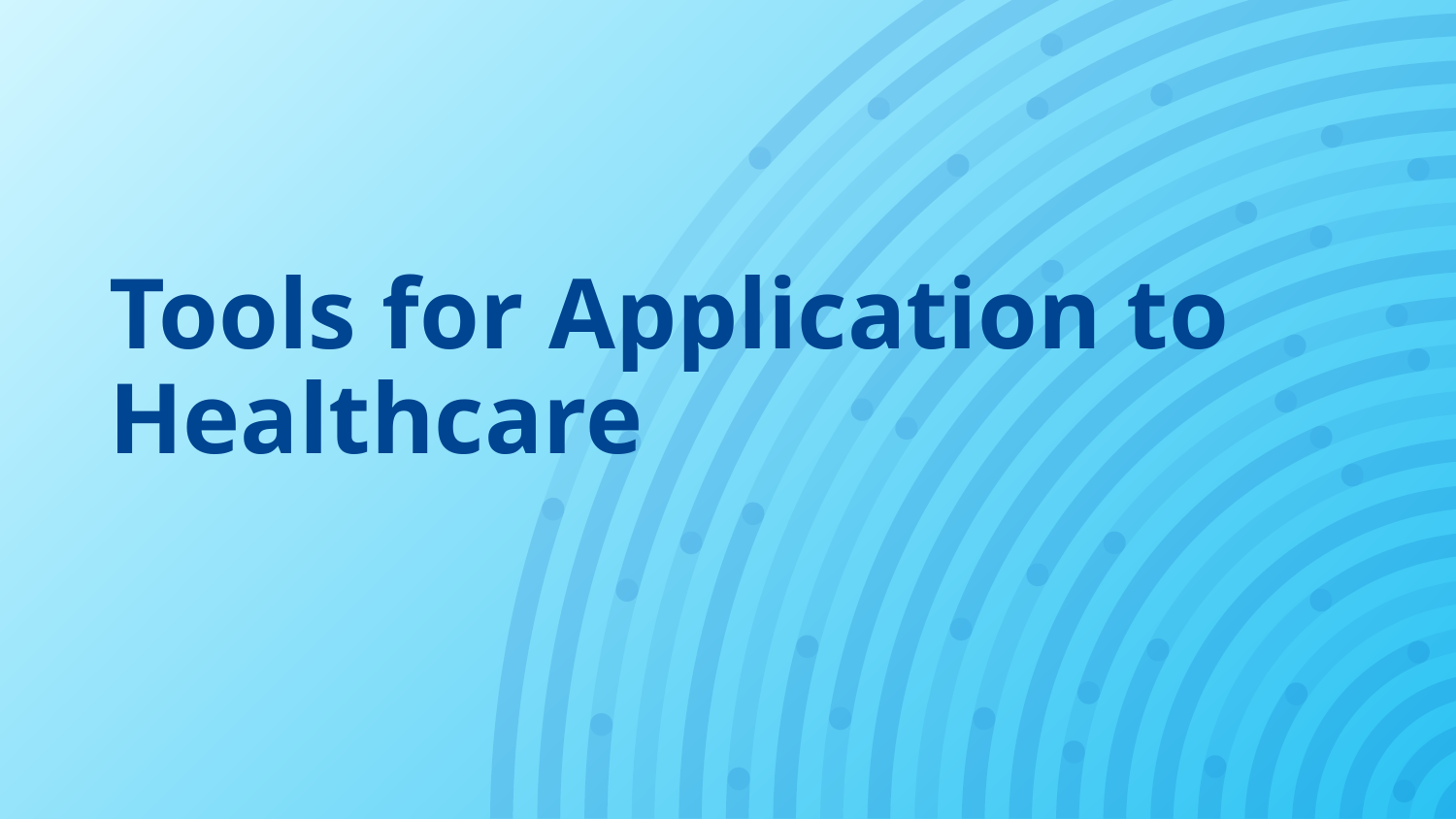

# Tools for Application to Healthcare

## Slide 28
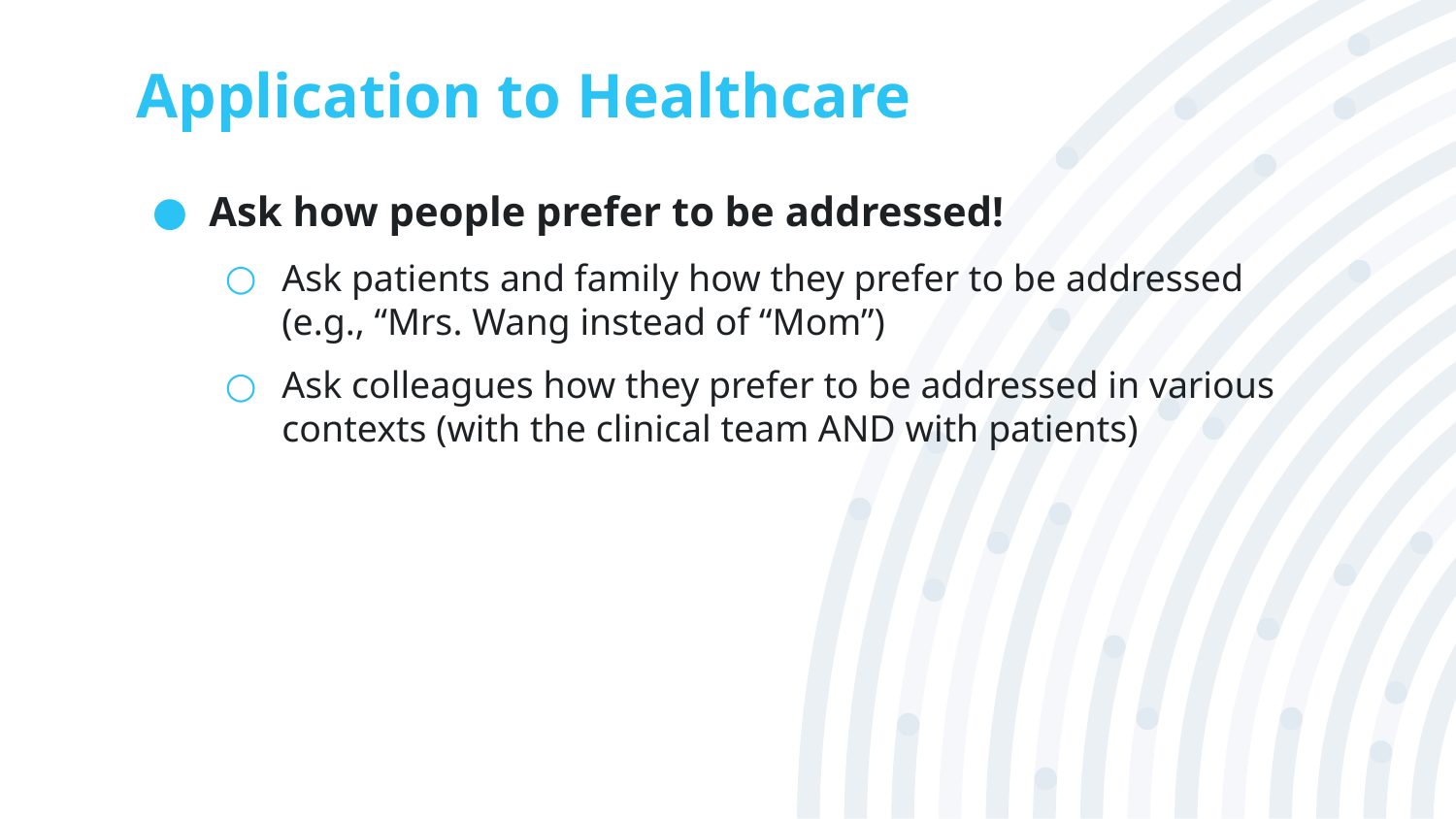

# Application to Healthcare
Ask how people prefer to be addressed!
Ask patients and family how they prefer to be addressed (e.g., “Mrs. Wang instead of “Mom”)
Ask colleagues how they prefer to be addressed in various contexts (with the clinical team AND with patients)

## Slide 29
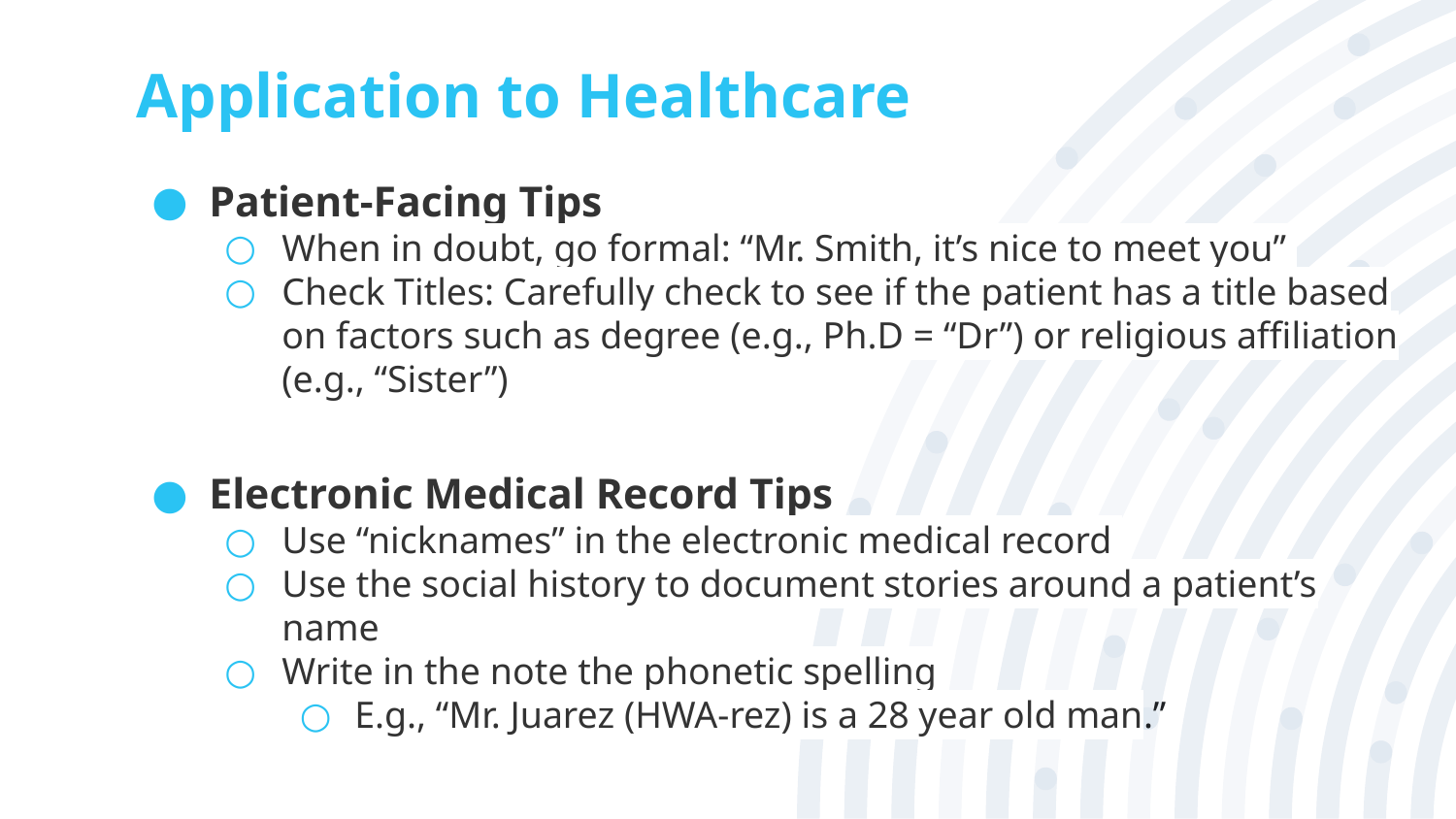

# Application to Healthcare
Patient-Facing Tips
When in doubt, go formal: “Mr. Smith, it’s nice to meet you”
Check Titles: Carefully check to see if the patient has a title based on factors such as degree (e.g., Ph.D = “Dr”) or religious affiliation (e.g., “Sister”)
Electronic Medical Record Tips
Use “nicknames” in the electronic medical record
Use the social history to document stories around a patient’s name
Write in the note the phonetic spelling
E.g., “Mr. Juarez (HWA-rez) is a 28 year old man.”

## Slide 30
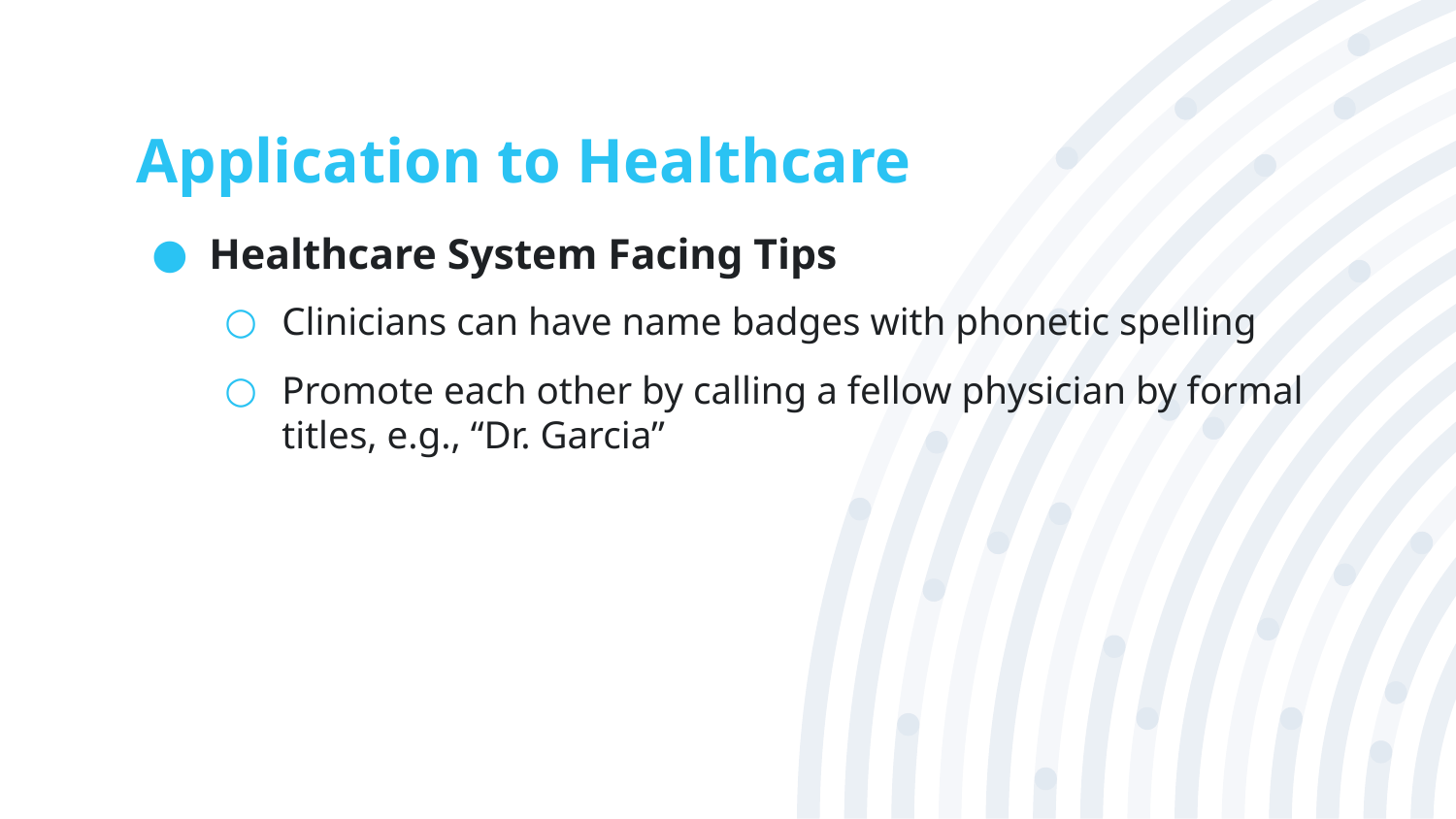

# Application to Healthcare
Healthcare System Facing Tips
Clinicians can have name badges with phonetic spelling
Promote each other by calling a fellow physician by formal titles, e.g., “Dr. Garcia”

## Slide 31
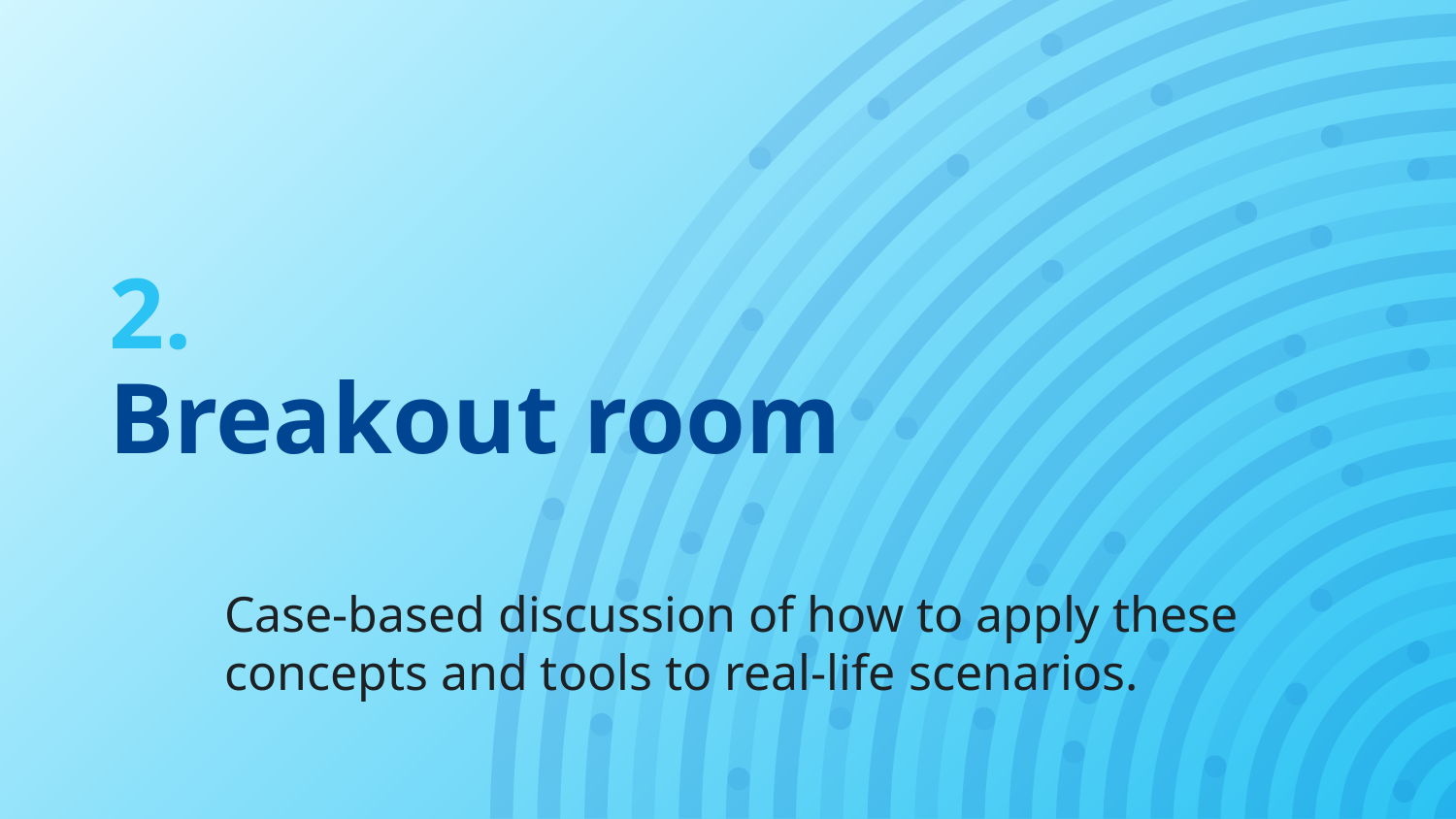

# 2.
Breakout room
Case-based discussion of how to apply these concepts and tools to real-life scenarios.

## Slide 32
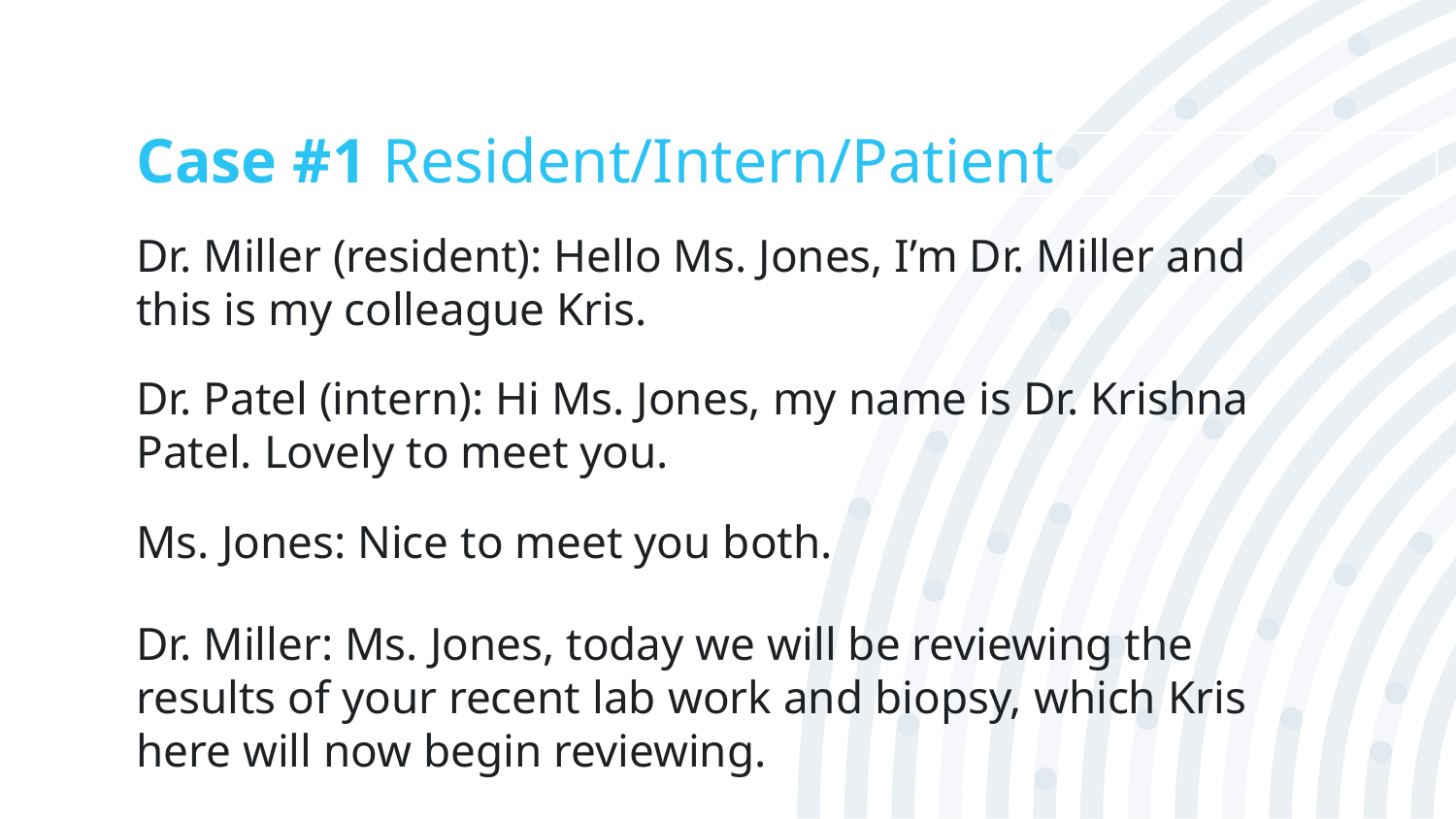

# Case #1 Resident/Intern/Patient
Dr. Miller (resident): Hello Ms. Jones, I’m Dr. Miller and this is my colleague Kris.
Dr. Patel (intern): Hi Ms. Jones, my name is Dr. Krishna Patel. Lovely to meet you.
Ms. Jones: Nice to meet you both.
Dr. Miller: Ms. Jones, today we will be reviewing the results of your recent lab work and biopsy, which Kris here will now begin reviewing.

## Slide 33
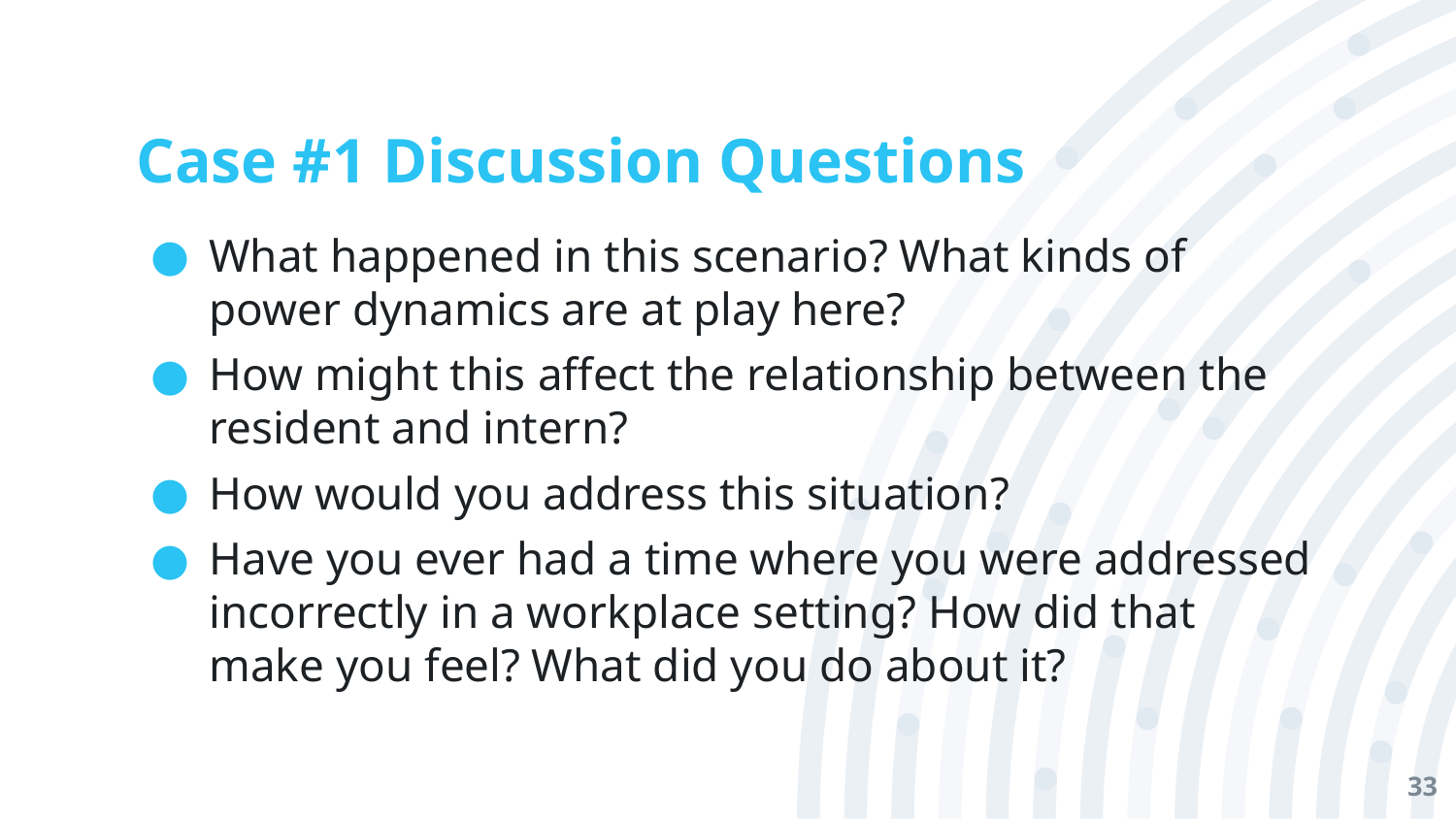

# Case #1 Discussion Questions
What happened in this scenario? What kinds of power dynamics are at play here?
How might this affect the relationship between the resident and intern?
How would you address this situation?
Have you ever had a time where you were addressed incorrectly in a workplace setting? How did that make you feel? What did you do about it?
33

## Slide 34
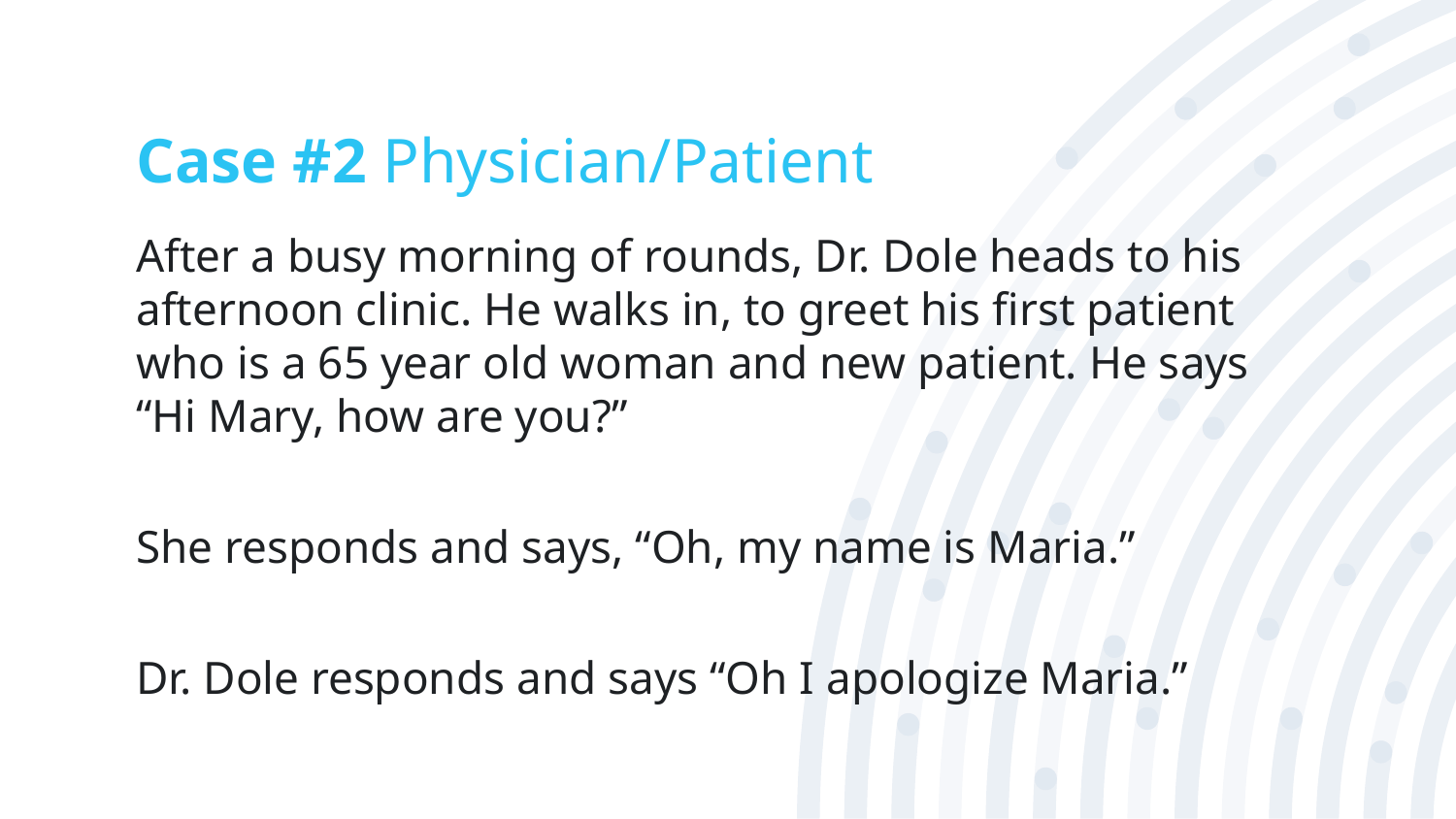

# Case #2 Physician/Patient
After a busy morning of rounds, Dr. Dole heads to his afternoon clinic. He walks in, to greet his first patient who is a 65 year old woman and new patient. He says “Hi Mary, how are you?”
She responds and says, “Oh, my name is Maria.”
Dr. Dole responds and says “Oh I apologize Maria.”

## Slide 35
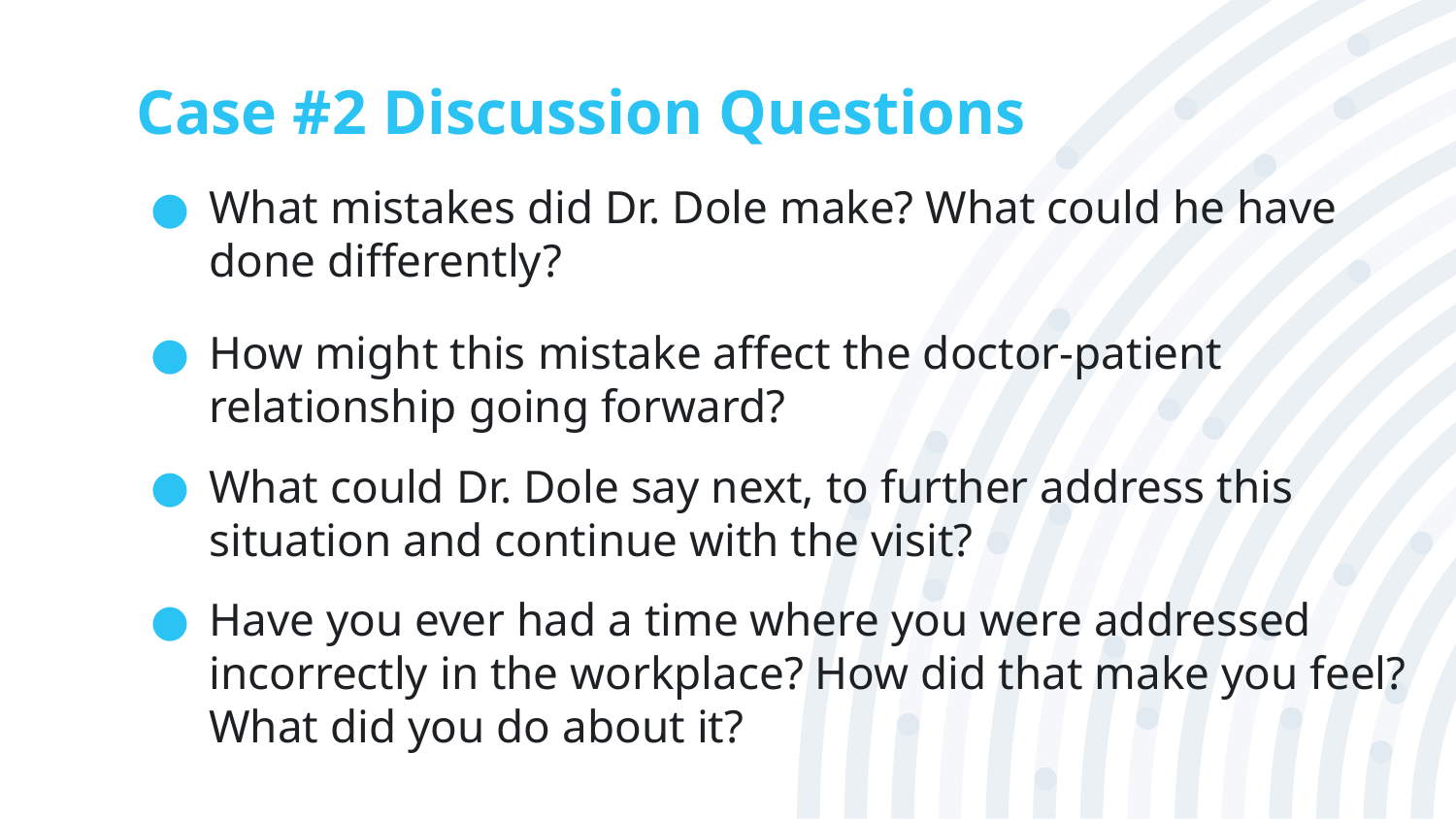

# Case #2 Discussion Questions
What mistakes did Dr. Dole make? What could he have done differently?
How might this mistake affect the doctor-patient relationship going forward?
What could Dr. Dole say next, to further address this situation and continue with the visit?
Have you ever had a time where you were addressed incorrectly in the workplace? How did that make you feel? What did you do about it?

## Slide 36
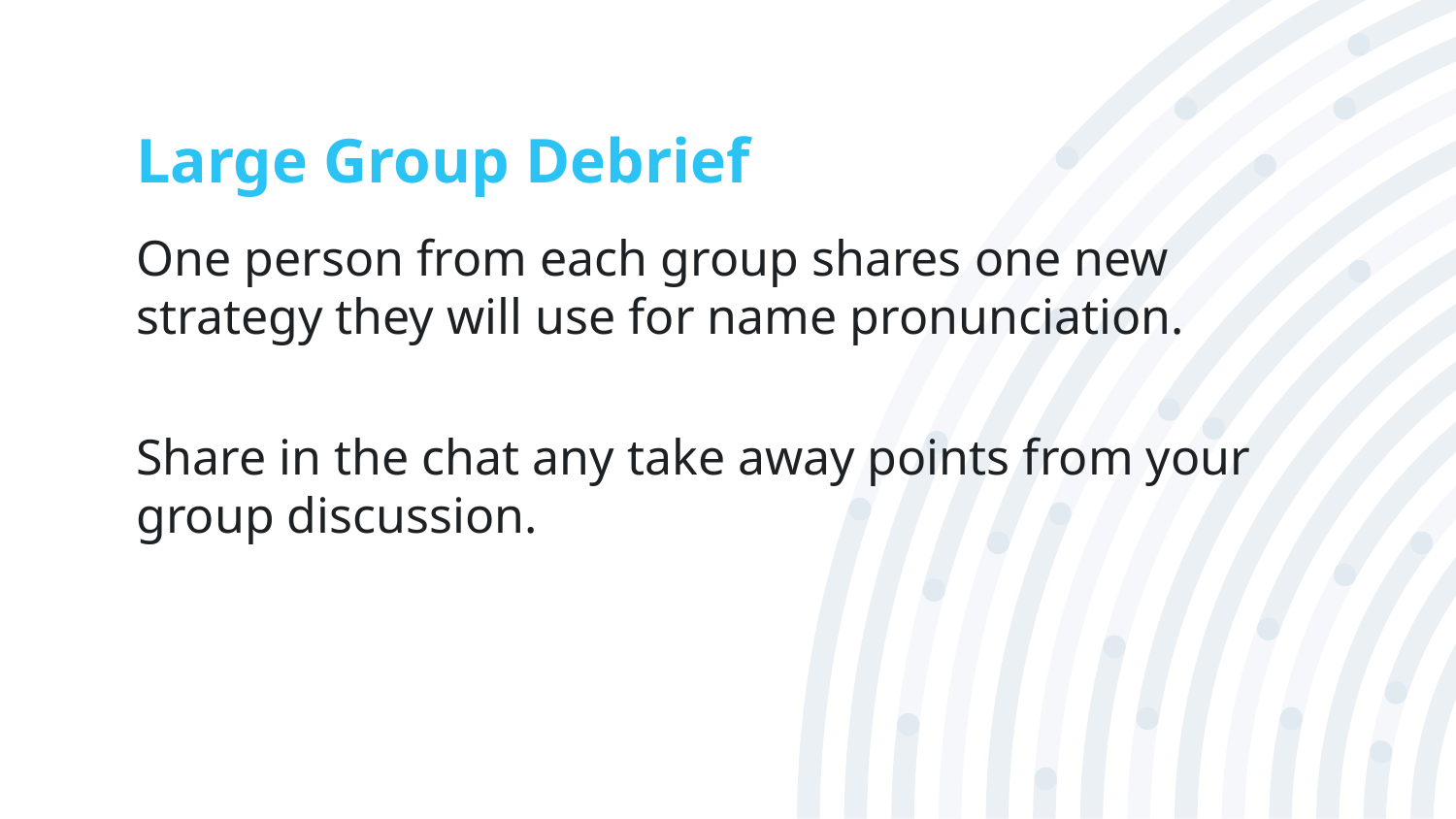

# Large Group Debrief
One person from each group shares one new strategy they will use for name pronunciation.
Share in the chat any take away points from your group discussion.

## Slide 37
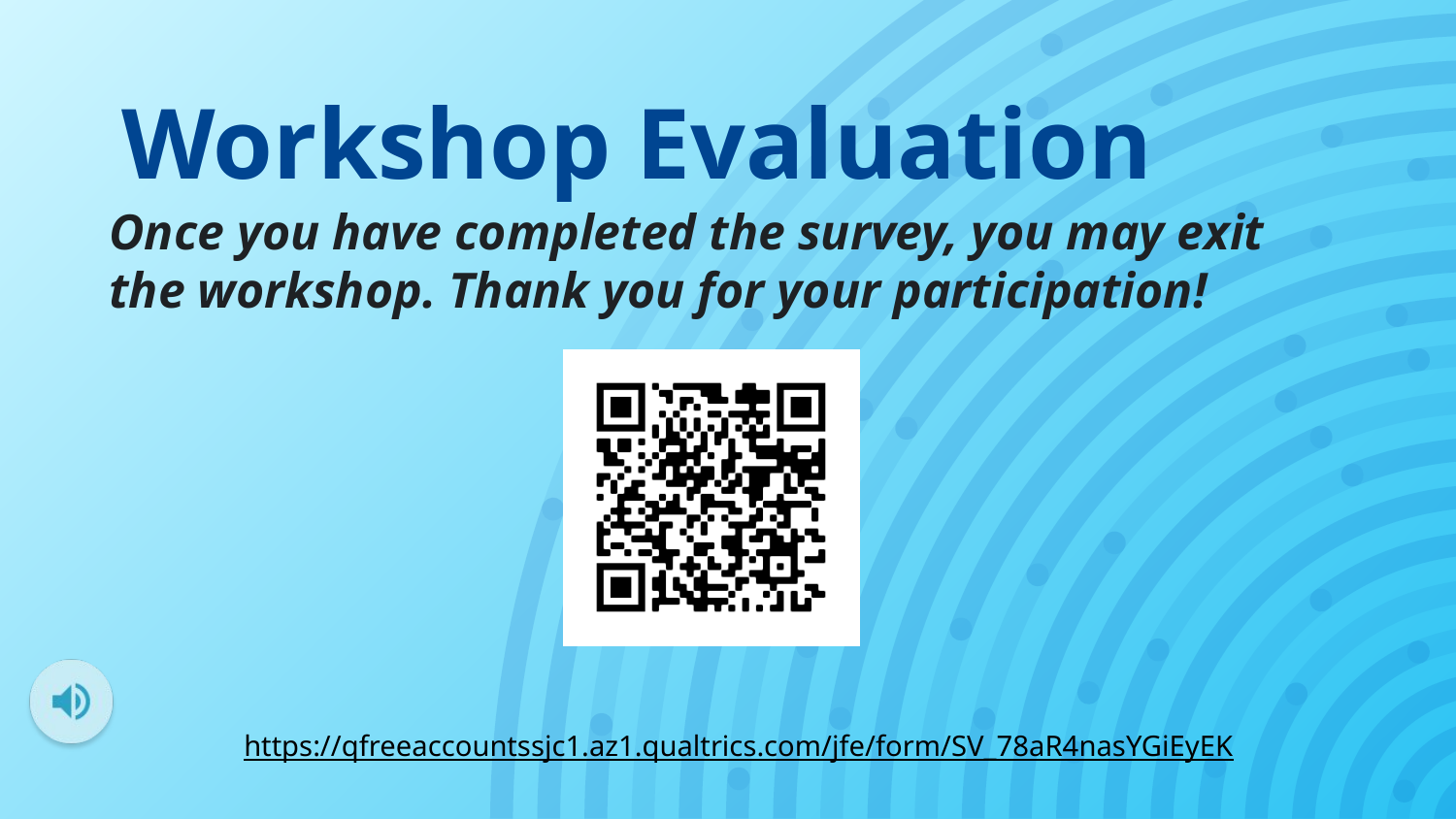

# Workshop Evaluation
Once you have completed the survey, you may exit the workshop. Thank you for your participation!
https://qfreeaccountssjc1.az1.qualtrics.com/jfe/form/SV_78aR4nasYGiEyEK

## Slide 38
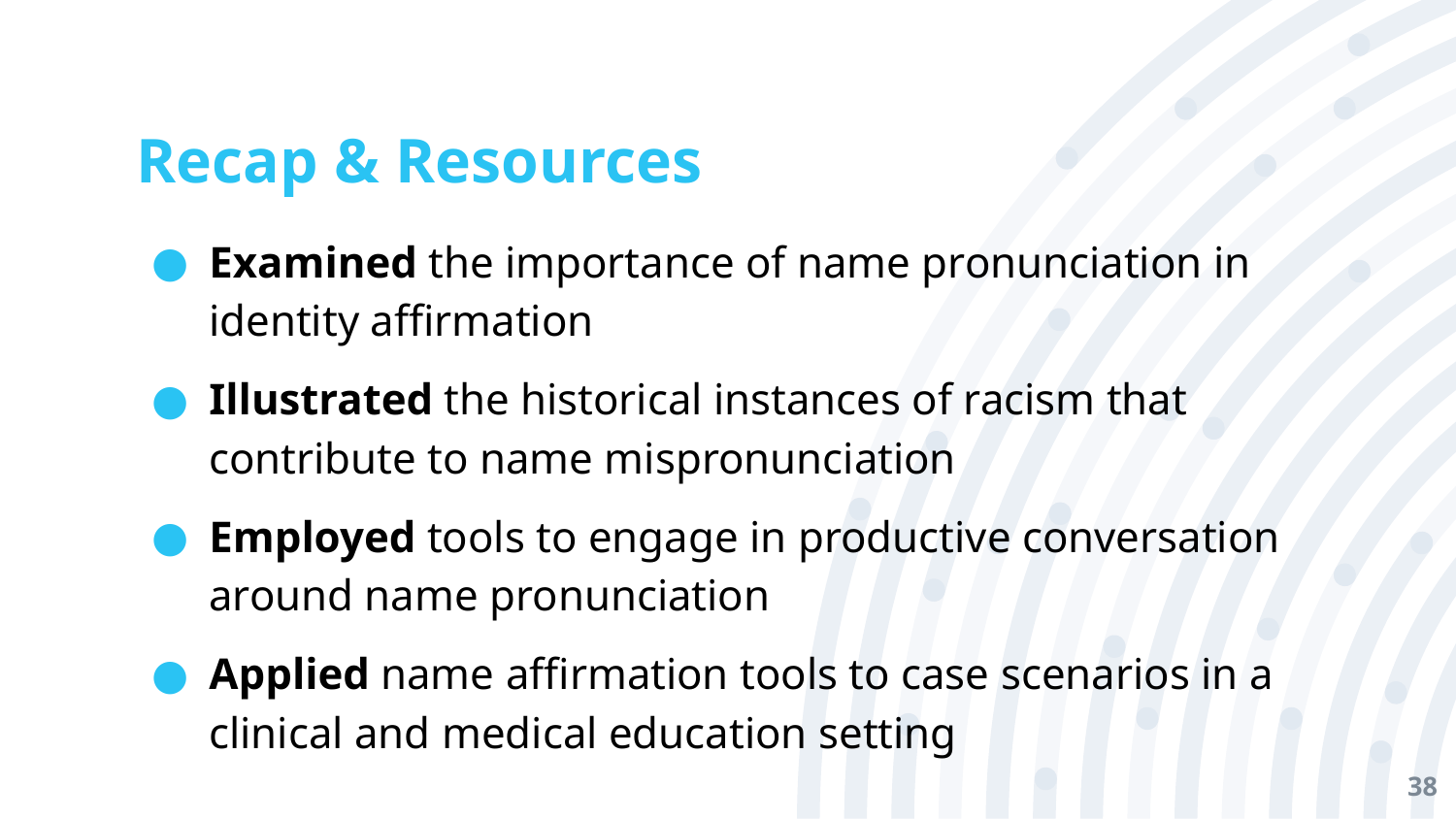

# Recap & Resources
Examined the importance of name pronunciation in identity affirmation
Illustrated the historical instances of racism that contribute to name mispronunciation
Employed tools to engage in productive conversation around name pronunciation
Applied name affirmation tools to case scenarios in a clinical and medical education setting
38

## Slide 39
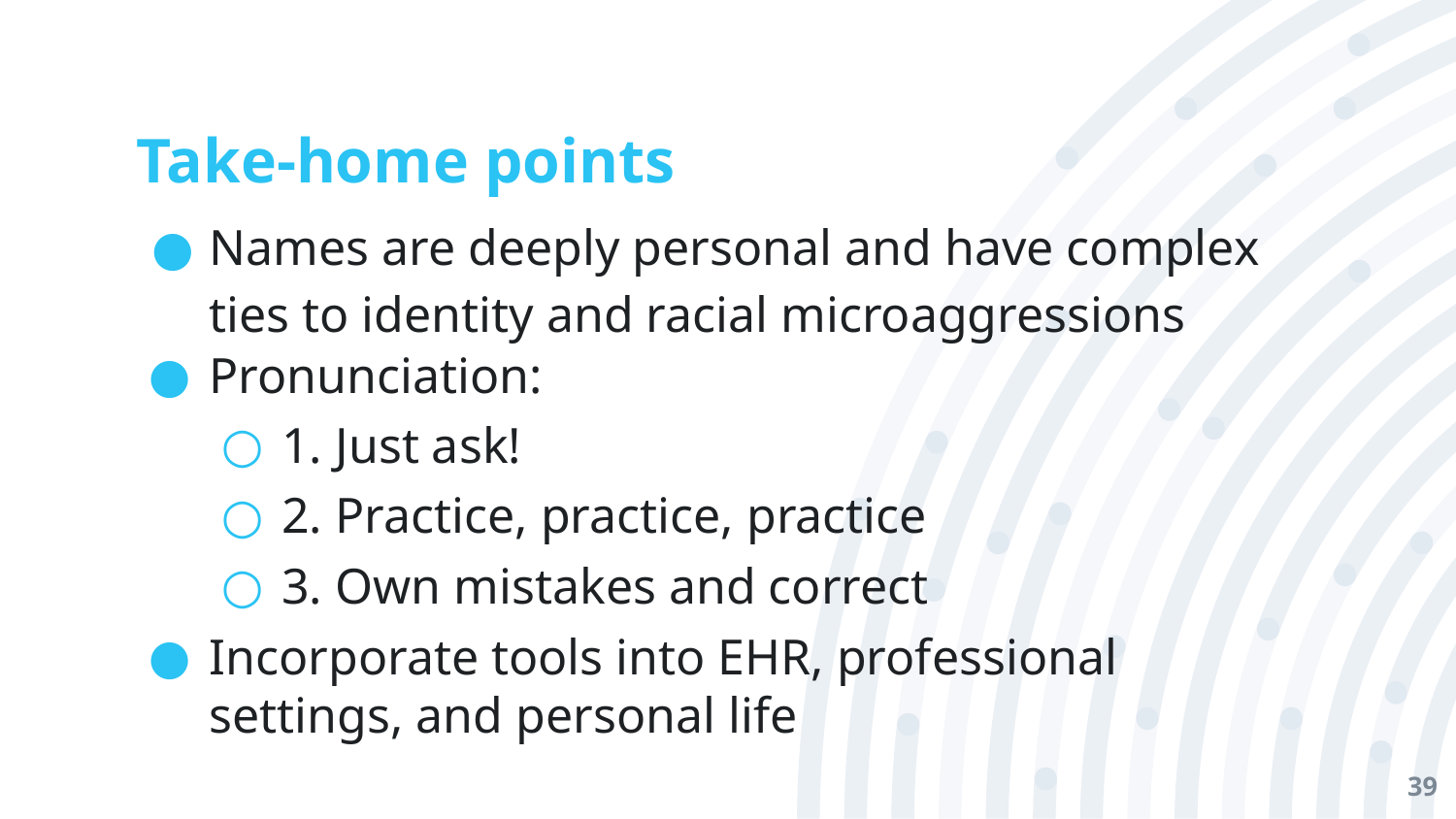

# Take-home points
Names are deeply personal and have complex ties to identity and racial microaggressions
Pronunciation:
1. Just ask!
2. Practice, practice, practice
3. Own mistakes and correct
Incorporate tools into EHR, professional settings, and personal life
39

## Slide 40
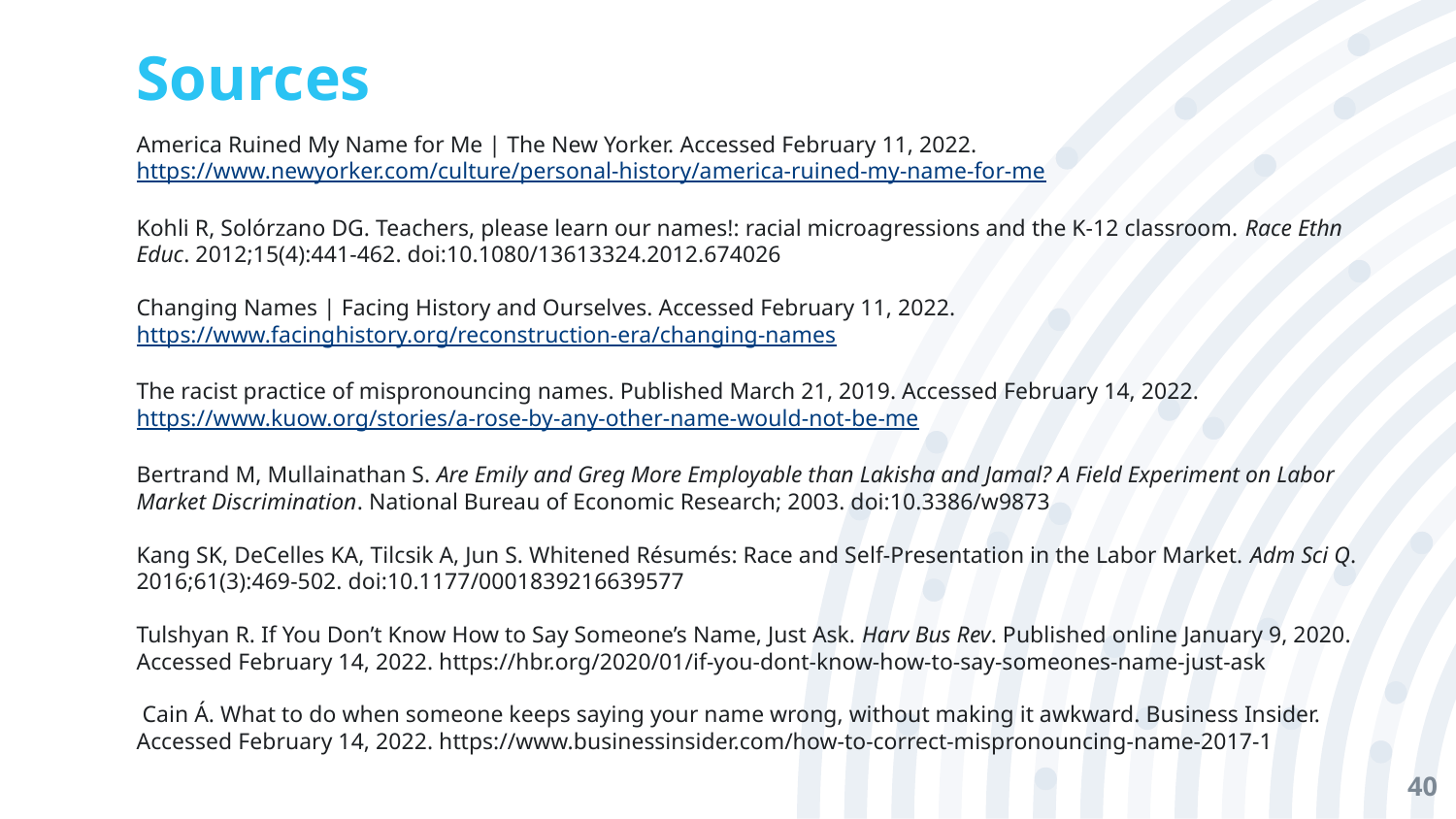

# Sources
America Ruined My Name for Me | The New Yorker. Accessed February 11, 2022. https://www.newyorker.com/culture/personal-history/america-ruined-my-name-for-me
Kohli R, Solórzano DG. Teachers, please learn our names!: racial microagressions and the K-12 classroom. Race Ethn Educ. 2012;15(4):441-462. doi:10.1080/13613324.2012.674026
Changing Names | Facing History and Ourselves. Accessed February 11, 2022. https://www.facinghistory.org/reconstruction-era/changing-names
The racist practice of mispronouncing names. Published March 21, 2019. Accessed February 14, 2022. https://www.kuow.org/stories/a-rose-by-any-other-name-would-not-be-me
Bertrand M, Mullainathan S. Are Emily and Greg More Employable than Lakisha and Jamal? A Field Experiment on Labor Market Discrimination. National Bureau of Economic Research; 2003. doi:10.3386/w9873
Kang SK, DeCelles KA, Tilcsik A, Jun S. Whitened Résumés: Race and Self-Presentation in the Labor Market. Adm Sci Q. 2016;61(3):469-502. doi:10.1177/0001839216639577
Tulshyan R. If You Don’t Know How to Say Someone’s Name, Just Ask. Harv Bus Rev. Published online January 9, 2020. Accessed February 14, 2022. https://hbr.org/2020/01/if-you-dont-know-how-to-say-someones-name-just-ask
 Cain Á. What to do when someone keeps saying your name wrong, without making it awkward. Business Insider. Accessed February 14, 2022. https://www.businessinsider.com/how-to-correct-mispronouncing-name-2017-1
40

## Slide 41
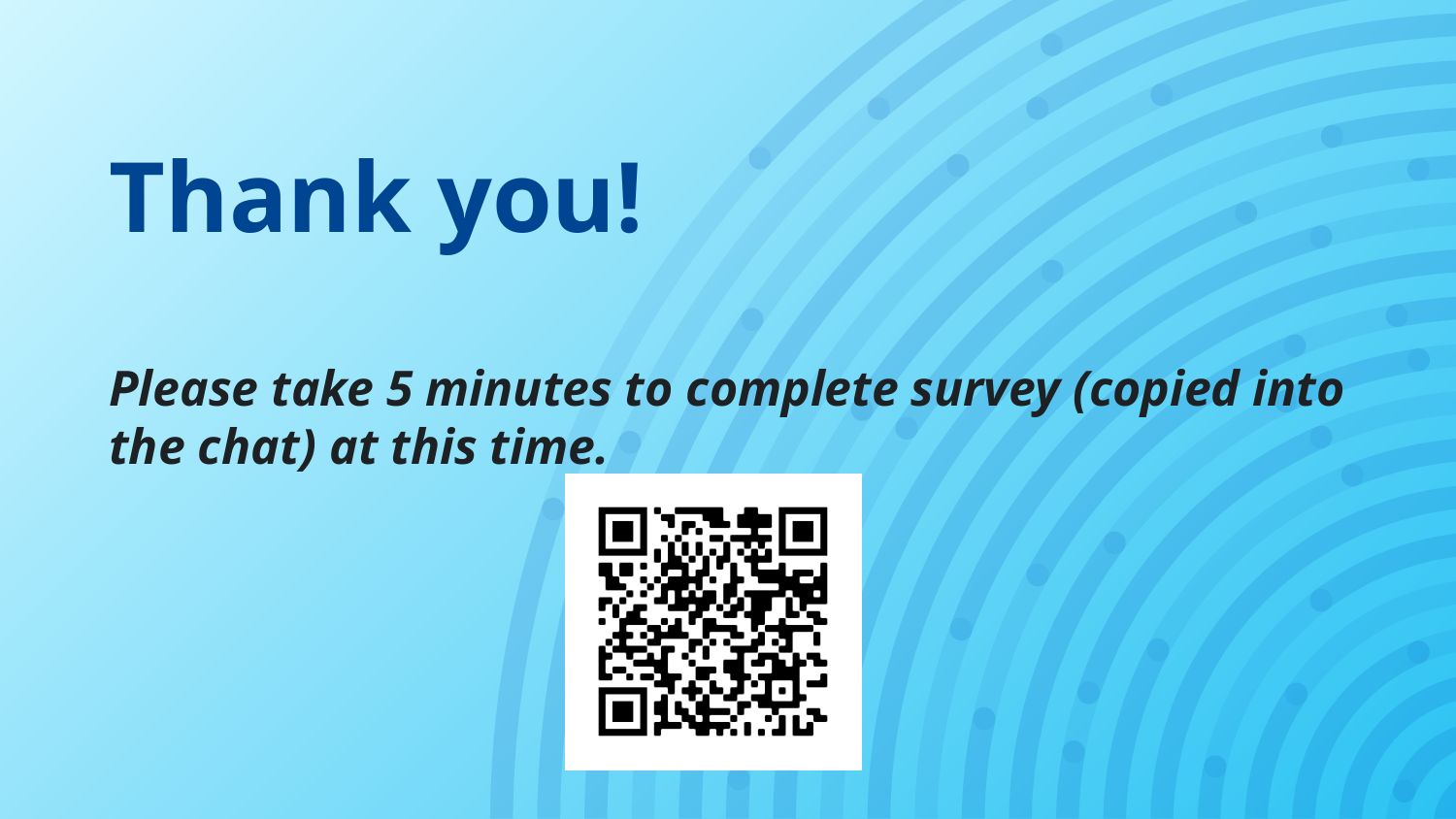

# Thank you!
Please take 5 minutes to complete survey (copied into the chat) at this time.
