# Supplementary material for: Say My Name: Understanding the Power of Names, Correct Pronunciation, and Personal Narratives
Source: MedEdPORTAL. 2022 Nov 29;18:11284. doi: 10.15766/mep_2374-8265.11284 (PMC9705275; doi:10.15766/mep_2374-8265.11284)
Supplement: Supplementary file 1 — Say My Name Presentation.pptxFacilitator Guide.docxParticipant Handout.docxPostworkshop Evaluation Form.docx [file mep_2374-8265.11284-s001.zip › B. Facilitator Guide.docx]

**Say My Name: Understanding the Power of Names, Correct Pronunciation, and Personal Narratives**

*Facilitator Guide*

**Educational objectives:**

By the end of this workshop, participants will be able to:

1. Examine the importance of name pronunciation in identity affirmation
2. Illustrate the historical instances of racism that contribute to name mispronunciation
3. Employ tools to engage in productive conversation around name pronunciation
4. Apply name affirmation tools to clinical setting, medical education, and workplace

**Detailed Agenda:**

General schedule: 75-minute version

| Introduction and objectives | Minute 0-5 (5 minutes) |
| --- | --- |
| Breakout Room #1: personal experience | Minute 5-15 (10 minutes) |
| Didactics   - Background - Poll - Historical context - Video 1 - Names and racism - Video 2 - Tools for pronunciation - Application zoom exercise - Application to healthcare | Minute 15-50 (35 mins)   - 3 minutes - 1 minute - 3 minutes - 3 minutes - 4 minutes - 1 minute - 15 minutes - 1 minute - 4 minutes |
| Breakout Room #2: case discussions | Minute 50-65 (15 minutes) |
| Large Group Debrief & Evaluations | Minute 65-75 (10 minutes) |

General schedule: 60-minute version

| Introduction and objectives | Minute 0-5 (5 minutes) |
| --- | --- |
| Breakout Room #1: personal experience | Minute 5-15 (10 minutes) |
| Didactics   - Background - Poll - Historical context - Video 1 - Names and racism - Video 2 - Tools for pronunciation - Application zoom exercise - Application to healthcare | Minute 15-45 (30 mins)   - 3 minutes - 1 minute - 3 minutes - 3 minutes - 4 minutes - 1 minute - 10 minutes - 1 minute - 4 minutes |
| Breakout Room #2: case discussions | Minute 45-55 (10 minutes) |
| Large Group Debrief & Evaluations | Minute 55-60 (5 minutes) |

**Supplies needed:**

1. PowerPoint Slides (Appendix A)
2. Participant Handout (Appendix C)
3. Postworkshop Evaluation (Appendix D)

**Introduction:**

- Ask participants to turn on cameras and mute microphones (facilitator should also mute all participants)
- Provide zoom instructions including Q&A preferences (chat, raise hand, etc)
- At least one facilitator should monitor the chat

**Polls:**

By either using the zoom function ‘Raise Hand’ or manually raising your hand, please raise your hand if you have ever mispronounced someone’s name

- Have you ever mispronounced someone’s name?
- Has your name ever been mispronounced?

Discuss poll results by acknowledging how prevalent name mispronunciation is on both sides

**Breakout Room #1:**

Instructions:

- At slide 5, please open 5 breakout rooms with participants randomly assigned
- Ask each group to assign one person to share before entering breakout rooms

Discussion Questions:

- Please introduce yourself with your name and an accompanying story, meaning of your name, or some aspect of how your name adds to your identity.
- Facilitator may provide the first example.

**Breakout room #2:**

Instructions:

- At slide 32, please open the same 5 breakout rooms as above

Cases and Discussion Questions:

Case #1

Dr. Miller (resident): Hello Ms. Jones, I’m Dr. Miller and this is my colleague Kris.

Dr. Patel (intern): Hi Ms. Jones, my name is Dr. Krishna Patel. Lovely to meet you

Ms. Jones: Nice to meet you both

Dr. Miller: Ms. Jones, today we will be reviewing the results of your recent lab work and biopsy, which Kris here will now begin reviewing.

- What happened in this scenario? What kinds of power dynamics are at play here?
- How might this affect the relationship between the resident and intern?
- How would you address this situation?
- Have you ever had a time where you were addressed incorrectly in a workplace setting? How did that make you feel? What did you do about it?

Case 1 facilitator discussion points:

- Power dynamics can further complicate name pronunciation. Often it becomes difficult to correct name pronunciation in a hierarchical structure.
- It is important to ask colleagues how they prefer to be addressed in personal as well as professional settings, as they may be different.
- Not using someone’s role or title can undermine them. This can affect the relationship between the resident and the intern, as well as between the intern and patient.
- Participants may identify other themes. These include shortening/altering names to make it easier for patients to pronounce, gender differences in using titles, varied personal preferences strategies for addressing mispronunciation.

Case #2

After a busy morning of rounds, Dr. Dole heads to his afternoon clinic. He walks in, to greet his first patient who is a 65 year old woman and new patient. He says “Hi Mary, how are you?”

She responds and says, “Oh, my name is Maria.”

Dr. Dole responds and says “Oh I apologize Maria.”

- What mistakes did Dr. Dole make? What could he have done differently?
- How might this mistake affect the doctor-patient relationship going forward?
- What could Dr. Dole say next, to further address this situation and continue with the visit?
- Have you ever had a time where you were addressed incorrectly by a professional you were seeing in consultation? How did that make you feel? What did you do about it?

Case 2 facilitator discussion points:

- The physician uses the incorrect name for the patient. This can lead to a lack of trust and impair the relationship between patient and physician.
- Instead, it is important to ask the patient what they prefer to be called. Starting with an open-ended question allows for the patient to include preferences including title and/or pronouns, and also allows them to express how preferences might vary with different groups (i.e. prefers one name in front of family and different name in front of colleagues).
- Remember to correct mispronunciation and own your mistakes. Facilitator and participants may refer to tools learned from the workshop.
- Participants may identify other themes and share personal stories.

**Large group debrief:**

- Ask one participant from each small group to share
- If time is limited, one small group from Case 1 and one small group from Case 2 can share

Facilitator debrief discussion points:

- Thank participants for sharing and address themes brought up by participants.
- Reiterate that names are deeply personal and have complex ties to identity and racial microaggressions.
- Highlight workshop tools shared by participants.
- Refer to Slides 38 and 39 in the PowerPoint (Appendix A) for main discussion points.

**Evaluations:**

- Allow time (~5 minutes) for participants to fill out postworkshop survey prior to concluding workshop
- Facilitator should provide link to postworkshop survey evaluation in the virtual chat and/or provide a QR code to collect responses <https://qfreeaccountssjc1.az1.qualtrics.com/jfe/form/SV_78aR4nasYGiEyEK>
- Review learning objectives and take-home points after participants have had time to complete postworkshop evaluation
