# Supplementary material for: Say My Name: Understanding the Power of Names, Correct Pronunciation, and Personal Narratives
Source: MedEdPORTAL. 2022 Nov 29;18:11284. doi: 10.15766/mep_2374-8265.11284 (PMC9705275; doi:10.15766/mep_2374-8265.11284)
Supplement: Supplementary file 1 — Say My Name Presentation.pptxFacilitator Guide.docxParticipant Handout.docxPostworkshop Evaluation Form.docx [file mep_2374-8265.11284-s001.zip › C. Participant Handout.docx]

**Say My Name: Understanding the Power of Names, Correct Pronunciation, and Personal Narratives**

*Participant Handout*

**Educational objectives:**

By the end of this workshop, participants will be able to:

1. Examine the importance of name pronunciation in identity affirmation
2. Illustrate the historical instances of racism that contribute to name mispronunciation
3. Employ tools to engage in productive conversation around name pronunciation
4. Apply name affirmation tools to clinical setting, medical education, and workplace

**Breakout Room #1 Discussion Question**

Please introduce yourself with your name and an accompanying story, meaning of your name, or some aspect of how your name adds to your identity.

**Breakout Room #2 Cases and Discussion Questions**

Case #1

Dr. Miller (resident): Hello Ms. Jones, I’m Dr. Miller and this is my colleague, Kris.

Dr. Patel (intern): Hi Ms. Jones, my name is Dr. Krishna Patel. Lovely to meet you

Ms. Jones: Nice to meet you both

Dr. Miller: Ms. Jones, today we will be reviewing the results of your recent lab work and biopsy, which Kris here will now begin reviewing.

- What happened in this scenario? What kinds of power dynamics are at play here?
- How might this affect the relationship between the resident and intern?
- How would you address this situation?
- Have you ever had a time where you were addressed incorrectly in a workplace setting? How did that make you feel? What did you do about it?

Case #2

After a busy morning of rounds, Dr. Dole heads to his afternoon clinic. He walks in, to greet his first patient who is a 65-year-old woman and new patient. He says “Hi Mary, how are you?”

She responds and says, “Oh, my name is Maria.”

Dr. Dole responds and says “Oh I apologize Maria.”

- What mistakes did Dr. Dole make? What could he have done differently?
- How might this mistake affect the doctor-patient relationship going forward?
- What could Dr. Dole say next, to further address this situation and continue with the visit?
- Have you ever had a time where you were addressed incorrectly by a professional you were seeing in consultation? How did that make you feel? What did you do about it?

**Name Pronunciation Tools**

[www.nameshout.com](http://www.nameshout.com)

[www.namecoach.com](http://www.namecoach.com)

[www.namez.com](http://www.namez.com)

<https://www.pronouncenames.com/>

<https://www.mynamemyidentity.org/>

**References**

<https://www.idealist.org/en/careers/name-correct-pronunciation>

<https://cos.gatech.edu/facultyres/Diversity_Studies/Bertrand_LakishaJamal.pdf>

<https://journals.sagepub.com/doi/pdf/10.1177/0001839216639577>

<https://www.youtube.com/watch?v=KXzVKmnukhA>

<https://hbswk.hbs.edu/item/minorities-who-whiten-job-resumes-get-more-interviews>

<https://wydaily.com/local-news/2020/08/03/ethnic-names-and-racism-how-some-monikers-might-not-get-you-hired/>

<https://www.wnycstudios.org/podcasts/takeaway/segments/role-names-racism-cultural-pride>

<https://www.facinghistory.org/reconstruction-era/changing-names>

<https://hbr.org/2020/01/if-you-dont-know-how-to-say-someones-name-just-ask>

<https://www.fastcompany.com/40553849/what-to-say-when-coworkers-wont-stop-mispronouncing-your-name>

<https://www.businessinsider.com/how-to-correct-mispronouncing-name-2017-1>

h[ttps://www.kevinmd.com/blog/2013/03/doctors-pronounce-names-patients-correctly.html](https://www.kevinmd.com/blog/2013/03/doctors-pronounce-names-patients-correctly.html)

<https://www.kevinmd.com/blog/2013/10/primer-addressing-patients.html>

<https://www.kevinmd.com/blog/2019/02/what-happened-to-the-first-name-in-medicine.html>

<https://www.newyorker.com/culture/personal-history/america-ruined-my-name-for-me>

<https://www.nber.org/papers/w9873>

<https://www.theguardian.com/world/2019/jan/17/minority-ethnic-britons-face-shocking-job-discrimination>
