# Supplementary material for: Say My Name: Understanding the Power of Names, Correct Pronunciation, and Personal Narratives
Source: MedEdPORTAL. 2022 Nov 29;18:11284. doi: 10.15766/mep_2374-8265.11284 (PMC9705275; doi:10.15766/mep_2374-8265.11284)
Supplement: Supplementary file 1 — Say My Name Presentation.pptxFacilitator Guide.docxParticipant Handout.docxPostworkshop Evaluation Form.docx [file mep_2374-8265.11284-s001.zip › D. Postworkshop Evaluation Form.docx]

**Say My Name: Understanding the Power of Names, Correct Pronunciation, and Personal Narratives**

*Post Workshop Evaluation*

**Educational objectives:**

By the end of this workshop, participants will be able to:

1. Examine the importance of name pronunciation in identity affirmation
2. Illustrate the historical instances of racism that contribute to name mispronunciation
3. Employ tools to engage in productive conversation around name pronunciation
4. Apply name affirmation tools to clinical setting, medical education, and workplace

Before attending the workshop, how likely were you to do the following

| Ask someone how to pronounce name | Extremely unlikely | Somewhat unlikely | Neither likely nor unlikely | Somewhat likely | Extremely likely |
| --- | --- | --- | --- | --- | --- |
| Correct someone for mispronouncing my name | Extremely unlikely | Somewhat unlikely | Neither likely nor unlikely | Somewhat likely | Extremely likely |
| Document the pronunciation of a name | Extremely unlikely | Somewhat unlikely | Neither likely nor unlikely | Somewhat likely | Extremely likely |

After attending the workshop, how likely are you to do the following

| Ask someone how to pronounce name | Extremely unlikely | Somewhat unlikely | Neither likely nor unlikely | Somewhat likely | Extremely likely |
| --- | --- | --- | --- | --- | --- |
| Correct someone for mispronouncing my name | Extremely unlikely | Somewhat unlikely | Neither likely nor unlikely | Somewhat likely | Extremely likely |
| Document the pronunciation of a name | Extremely unlikely | Somewhat unlikely | Neither likely nor unlikely | Somewhat likely | Extremely likely |

To what extent do you agree or disagree with the following

| I am more aware of the importance of name pronunciation | Strongly disagree | Somewhat disagree | Neither agree nor disagree | Somewhat agree | Strongly agree |
| --- | --- | --- | --- | --- | --- |
| I learned about historical context of racism that contributes to name mispronunciation | Strongly disagree | Somewhat disagree | Neither agree nor disagree | Somewhat agree | Strongly agree |
| I will apply the name pronunciation and affirmation tools I learned today | Strongly disagree | Somewhat disagree | Neither agree nor disagree | Somewhat agree | Strongly agree |

What were strengths of the workshop?

How can this workshop be improved?

What are two things you learned from this workshop that you will apply?

What are potential barriers to applying what you have learned?

What is your profession? (Select all that apply)

- Medical student
- Resident
- Fellow
- Clinician researcher
- Clinician faculty
- Business
- Education
- Write in

Race/ethnicity (select all that apply)

- Black or African American
- American Indian or Alaskan Native
- White or Caucasian
- Hispanic, Latinx, or Spanish origin
- Asian
- Write in

Gender identity

- Male
- Female
- Non-binary / third gender
- Write in

Age

We thank you for your time spent taking this survey.
Your response has been recorded.

<https://qfreeaccountssjc1.az1.qualtrics.com/jfe/form/SV_78aR4nasYGiEyEK>
